# Participation of the Halogens in Photochemical Reactions in Natural and Treated Waters

**DOI:** 10.3390/molecules22101684

**Published:** 2017-10-13

**Authors:** Yi Yang, Joseph J. Pignatello

**Affiliations:** Department of Environmental Sciences, The Connecticut Agricultural Experiment Station, 123 Huntington St., P.O. Box 1106, New Haven, CT 06504-1106, USA; yangyihit@hotmail.com

**Keywords:** hydroxyl radical, sulfate radical, photocatalysis, atmospheric aerosols, reactive oxygen species, reactive halogen species, advanced oxidation processes, dissolved natural organic matter, halogenation, reclaimed waters

## Abstract

Halide ions are ubiquitous in natural waters and wastewaters. Halogens play an important and complex role in environmental photochemical processes and in reactions taking place during photochemical water treatment. While inert to solar wavelengths, halides can be converted into radical and non-radical reactive halogen species (RHS) by sensitized photolysis and by reactions with secondary reactive oxygen species (ROS) produced through sunlight-initiated reactions in water and atmospheric aerosols, such as hydroxyl radical, ozone, and nitrate radical. In photochemical advanced oxidation processes for water treatment, RHS can be generated by UV photolysis and by reactions of halides with hydroxyl radicals, sulfate radicals, ozone, and other ROS. RHS are reactive toward organic compounds, and some reactions lead to incorporation of halogen into byproducts. Recent studies indicate that halides, or the RHS derived from them, affect the concentrations of photogenerated reactive oxygen species (ROS) and other reactive species; influence the photobleaching of dissolved natural organic matter (DOM); alter the rates and products of pollutant transformations; lead to covalent incorporation of halogen into small natural molecules, DOM, and pollutants; and give rise to certain halogen oxides of concern as water contaminants. The complex and colorful chemistry of halogen in waters will be summarized in detail and the implications of this chemistry for global biogeochemical cycling of halogen, contaminant fate in natural waters, and water purification technologies will be discussed.

## 1. Introduction

Halide ions are ubiquitous in natural waters. Ordinary levels of halides in seawater are 540 mM chloride, 0.8 mM bromide, and 100–200 nM iodide [1,2]. Halide levels range downward in estuaries and upward in saltier water bodies relative to typical seawater levels. Surface fresh water and groundwater may contain up to 21 mM chloride and 0.05 mM bromide [1], with higher levels in some places. Even though the halides themselves do not absorb light in the solar region, in nature they provide far more than just background electrolytes—they participate in a rich, aqueous-phase chemistry initiated by sunlight that has many implications for dissolved natural organic matter (DOM) processing, fate and toxicity of organic pollutants, and global biogeochemical cycling of the halogens.

Advanced oxidation processes (AOPs) employing solar, visible, or ultraviolet light have been used or are under study for removal of organic pollutants from reclaimable waters, such as industrial wastewater, petrochemical produced waters, municipal wastewater, and landfill leachates, in order to meet agricultural, residential, business, industrial, or drinking water standards. While generalizations are difficult, such waters often contain moderate-to-very-high halide ion concentrations, as well as high concentrations of other photochemically important solutes like carbonate that can impact halogen chemistry [1].

This review aims to summarize the reactions of halides and their daughter products and offer insight into their effects on photochemical transformations taking place in water. Halides can undergo sensitized photolysis and react with many secondary photoproducts to produce reactive halogen species (RHS) that can participate in a variety of reactions with DOM and anthropogenic compounds, including oxidation and incorporation of halogen. These reactions are described and discussed. Extensive tabulations of rate constants for relevant reactions or RHS generation and decay have been collected for the convenience of the reader in Supplementary Section Table S1. Halides, and the RHS derived from them, affect the concentrations of photogenerated reactive oxygen species (ROS) and other reactive species; influence the photobleaching of DOM; alter the rates and products of pollutant transformations; lead to covalent incorporation of halogen into small natural molecules, dissolved natural organic matter, and pollutants; and give rise to certain halogen oxides of concern as water contaminants. The concentrations of halides is an important consideration in water treatment because halides can scavenge desired reactive oxidants and lead to unwanted halogenated byproducts. The identity of the halogen substituent(s) is critical because toxicity ordinarily increases in the order Cl < Br < I for compounds of similar structure [3,4].

Halogen reactions in the atmosphere have been well studied in relation to ozone chemistry [5]. This article will not discuss gas phase reactions or surface reactions in the atmosphere, a topic recently addressed in a comprehensive review [5]; however, it will cover relevant reactions that occur in the liquid phase or at the air-liquid interface of atmospheric aerosols. A number of important reactions that take place on snow, ice, and solid microparticles actually occur on or within a surface liquid layer that is often rich in salts [6]. Compared to bulk natural waters, aerosol liquid phases can reach lower pH, and the evidence supports altered rates and/or unique chemical reactions close to the air-liquid interface.

## 2. Sources and Speciation of RHS Produced from Halide Ions

**Scheme 1:**
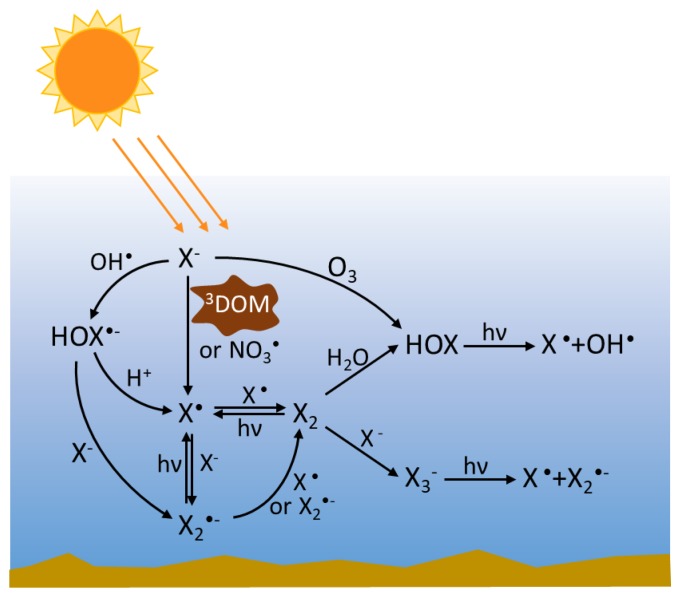
Generation of RHS in waters through the action of sunlight.

Reactive halogen species are generated by sensitized photochemical reactions or by reaction of halides with other oxidants of a photochemical origin. Halogen interconversion reactions are dealt with in detail. Scheme 1 provides an overview.

### 2.1. Sensitized Photolysis

Halide ions in aqueous solution have absorption edges below ~260 nm and therefore do not photolyze at solar wavelengths. However, recent studies indicate that photo-sensitization by DOM may be an important source of RHS in natural waters [7,8]. Irradiation of DOM with solar light generates a short-lived excited singlet state (^1^DOM*) that can relax to the ground state or intersystem crosses (ISC) to a much longer-lived excited triplet state (^3^DOM*). ^3^DOM* is a mixture of excited triplet states of diverse structures with energies ranging from 94 kJ·mol^−1^ to above 250 kJ·mol^−1^ [9]. While the nature of the chromophoric groups of DOM giving rise to triplet states is not known for certain, it has been said that aromatic ketones and other carbonyl-containing groups (e.g., coumarin and chromone moieties) are candidates for production of the high-energy triplet states of DOM [10]. The steady-state concentration of ^3^DOM* is estimated to be 10^−14^ to 10^−12^ M, depending on light intensities, [DOM] and [O_2_] [10] and, undoubtedly, the nature of DOM in the water parcel.

^3^DOM* is a known precursor of photochemically-produced reactive oxygen species (ROS) such as singlet oxygen (^1^O_2_) and hydroperoxyl/superoxide (HO_2_^•^/O_2_^−•^, p*K*_a_ = 4.88), and is a suspected precursor of hydroxyl (HO^•^). In addition, ^3^DOM* also can engage in triplet energy transfer or oxidation reactions with itself and with other solutes. It has been shown that ^3^DOM* can oxidize or reduce various organic compounds [11], and that model triplet ketone sensitizers with similar reactivity as ^3^DOM* can oxidize CO_3_^2−^ to CO_3_^−•^, NO_2_^−^ to NO_2_^•^ [12], etc.

The question arises whether ^3^DOM* can oxidize halide ions. The standard reduction potential of ^3^DOM* obtained in different studies of terrestrial and freshwater NOM reference standards is estimated to be “centered near 1.64 V” [10] and about 1.6–1.8 V [8]. The estimated one-electron reduction potentials of the halogens ${E{}^{\circ}}_{X\cdot {/X}^{-}}$ are 2.59 V (Cl), 2.04 V (Br), and 1.37 V (I) in water [13]. These values are about 0.4–0.5 V lower in polar organic solvents—an important consideration because DOM exists as supramolecular aggregates and colloids, in which the electric field in the vicinity of the chromophoric site may be somewhere in between water and polar organic solvents. It thus appears that bromide and iodide, and possibly chloride, are potentially susceptible to one-electron oxidation by ^3^DOM*.

Jammoul et al. [7] found that the triplet excited state of benzophenone, which can be regarded as a surrogate for aromatic carbonyl compounds in seawater DOM, can oxidize halide ions to X_2_^−•^, Reaction (1):

(1) $${\left[{\left(C_{6}H_{5}\right)}_{2}C=O\right]}^{3*}+2X^{-}\overset{\mathrm{hv}(355\;\mathrm{nm})}{\to }{\left[{\left(C_{6}H_{5}\right)}_{2}C-O\right]}^{-•}+X_{2}{}^{-•}$$

The rate constant for Reaction (1) follows the order, I^−^ (~8 × 10^9^) > Br^−^ (~3 × 10^8^) > Cl^−^ (<1 × 10^6^ M^−1^ s^−1^) which is consistent with the order in their reduction potential. The triplet state of anthraquinone derivatives was observed to oxidize bromide and chloride [12,14].

**Scheme 2:**
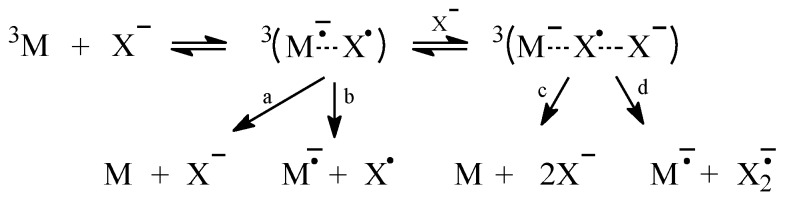
Proposed pathways of sensitized oxidation of halide ions in water.

Building on previous theory [15], Loeff et al. [12] modeled reactions sensitized by simple organic compounds according to Scheme 2.

According to this model, halide ion reacts with the triplet excited state (^3^M) to form a charge-transfer binary exciplex, ^3^(M^−^ --- X), or, at higher halide concentrations, the ternary exciplex, ^3^(M^−^---X --- X^−^). Both the binary and ternary exciplexes can decay to the ground state (paths a or c) or dissociate to the radical pair (paths b or d). The ternary exciplex has a lower tendency than the binary exciplex to decay to the ground state because it has weaker spin-orbit coupling of the incipient radical. Therefore, the ternary exciplex more favorably dissociates to the radical products, M^−•^ and X_2_^−•^. In seawater, the mixed dihalogen radical anion, BrCl^−•^, is expected to predominate, since bromide is more readily oxidized [16], while chloride is more abundant.

Comparing artificial seawater with ionic strength controls (NaClO_4_), Parker and Mitch [8] report that ^3^DOM* contributes to RHS formation, which, in turn, affects the oxidation of certain added organic compounds. Using a series of radical quenching agents, they found a strong linear correlation between the observed rate constant for degradation of the marine algal toxin domoic acid sensitized by a DOM reference standard, and the same rate constant sensitized by bromoacetophenone which generates Br^•^ upon photolysis. In support of Scheme 2 for DOM, the researchers found that chloride enhances bromination in samples containing bromide.

In summary, Scheme 2 has been able to rationalize the behavior of simple sensitizer molecules. Even though the scant data available on DOM is consistent with it, it is far from being “established” for DOM and further studies are called for.

### 2.2. Oxidation of Halide Ions by Secondary Photo-Products

Sunlight directly or indirectly produces OH^•^, ozone (O_3_), ^1^O_2_, HO_2_^•^/O_2_^−•^, and hydrogen peroxide (H_2_O_2_) in natural waters. Such ROS are important in many AOPs, as well. Halide ions are susceptible to oxidation by several of these ROS.

One of the most important is HO^•^. Hydroxyl originates from direct photolysis of H_2_O_2_, NO_3_^−^, NO_2_^−^, DOM, and dissolved iron species, and can also be produced by (dark) Fenton-type reactions of H_2_O_2_ catalyzed by redox-switchable transition metal ions, especially Fe. Which of these sources are most important depends on local conditions and is difficult to ascertain in most situations. The exact mechanism of HO^•^ generation from DOM has been the subject of debate for many years, without consensus [17,18,19]. Hydroxyl reacts with halides via the adduct HOX^−•^ to form the corresponding halogen and dihalogen radicals:

(2) $$X^{-}+HO^{•}\overset{\;}{⇄}HOX^{-•}\overset{H^{+},-H_{2}O}{⇄}X^{•}\overset{X^{-}}{⇄}X_{2}{}^{-•}$$

Reaction (2) is fast, reversible, and dependent on [X^−^] and [H^+^] [20]. Reactions with bromide and iodide lie far to the right at any normal environmental pH, while the oxidation of chloride to Cl^•^ and Cl_2_^−•^ is favorable only under acidic conditions and comparatively high halide concentrations. For example, at pH 3, oxidation of chloride is significant whenever [Cl^−^] is much above a few millimolar [21]. However, oxidation of chloride can be important in aerosols, where the pH can be as low as 2. Bromide and iodide are important OH^•^ scavengers in seawater [17]. Scavenging of OH^•^ does not necessarily protect other solute molecules from oxidation, as the resulting RHS are themselves strong oxidants, albeit more selective (see Section 4).

Ozone is an important component of the troposphere due to the action of sunlight on nitrogen oxides and organic vapors. Ground-level ozone concentrations can be appreciable especially in urban and industrial areas [22,23,24]. The reaction of ozone with halide initially produces hypohalite or hypohalous acid (XO^−^/XOH; p*K*_a,HOCl_ = 7.82 (0 °C), 7.54 (25 °C); p*K*_a,HOBr_ = 8.55; p*K*_a,HOI_ = 10.5), via a transient halo-ozonide intermediate [25]:

(3) $$X^{-}+O3\overset{}{⇄}X-OOO^{-}\overset{H_{2}O}{\to }XOH+O_{2}+OH^{-}$$

The observed rate constants for overall Reaction (3) differ by more than twelve orders of magnitude among the halogens (*k*_Cl−_ < 3 × 10^−3^ M^−1^ s^−1^; *k*_Br−_ = 258 M^−1^ s^−1^; *k*_I−_ = 1.2 × 10^9^ M^−1^ s^−1^) [25,26]. Given normal seawater halide concentrations, the ratio of *rates* for ozone oxidation of iodide, bromide, and chloride is thus approximately 2300:130:1.

Reaction with O_3_ is suggested to be a principal source of bromo- and iodo-RHS in seawater [27]. Since HOI can react with Br^−^ and Cl^−^ to form molecular bromine and chlorine species and regenerate I^−^ (see Section 2.4), iodide has been implicated as a catalyst for volatilization of bromine and chlorine from marine aerosol microdroplets [28].

Halides react only slowly with ^1^O_2_; second order rate constants are 1 × 10^3^ for Cl^−^ (in D_2_O); < 1 × 10^6^ for Br^−^ (in acetone/bromobenzene solution), and 8.7 × 10^5^ M^−1^ s^−1^ for I^−^ (in water)—too slow to compete with physical quenching of ^1^O_2_ by water (2.5 × 10^5^ s^−1^ [29]). The halides do not react with HO_2_^•^/O_2_^−•^ in water at environmentally significant rates. A few other oxidation reactions of halides are important in aerosol systems (Section 2.3).

### 2.3. Heterogeneous Reactions Leading to RHS

Halides also participate in both dark and actinic heterogeneous chemistry in or on atmospheric particles [30,31]. Atmospheric aerosols broadly encompass polar stratospheric cloud particles of nitric and sulfuric acid hydrates; cloud particles of water ice; soil dusts; marine boundary layer aerosols consisting of sea salts; secondary organic aerosols resulting from oxidation of biogenic compounds in the troposphere; combustion aerosols of fuels and biomass; and inorganic ammonium salt aerosols. Many of these types of particles are relevant here, either because they are aqueous liquids, or because their surfaces are coated with aqueous films that exist due to the high salt levels which attract water.

Reactions of halides in aerosol liquids can be qualitatively and quantitatively different from reactions in terrestrial waters owing to their small size and the significance of gas-particle interfacial phenomena [30]:

(i) The pH is often more acidic in the bulk liquid phase of aerosols than in terrestrial water bodies. By contrast, the air-liquid interface can be significantly more basic than the bulk aerosol phase; for example, it is known that the pH is 7 at the surface of bulk water at pH 3 [32].
(ii) The heavier halide ions (Br^−^, I^−^) concentrate at the air-liquid interface. Evidence exists for unique chemical reactions close to the air-liquid interface [33].
(iii) Particles may become depleted in bromide and iodide with respect to chloride, so that the chemistry can change over time.
(iv) Reactions may be sensitive to humidity which governs film thickness.

Halide conversion to RHS on atmospheric aerosols is initiated mainly by reactions with HO^•^, O_3_, nitrate radical (NO_3_^•^), and N_2_O_5_. Their reactions with HO^•^ and O_3_ are given in Reactions (2) and (3) above. Pratt et al. [6] found that Br_2_ is generated on arctic fresh snow by oxidation of Br^−^ by HO^•^ formed by photolysis of NO_2_^−^ or H_2_O_2_ within the quasi-brine layer on the snow surfaces. The volatilized Br_2_ is postulated to get pumped by the wind into the troposphere where it contributes to the episodic depletion of tropospheric ozone during the Arctic springtime.

Nitrate radical, which originates from oxidation of nitrogen dioxide (NO_2_) by ozone [34], is an important atmospheric free radical, especially at night. It rapidly oxidizes aerosol halides (Reaction 4) [35,36]:

(4) $$X^{-}+{NO_{3}}^{•}\overset{\;}{\to }X^{•}+NO_{3}{}^{-}k_{Cl-}=3.5\times 10^{8}M^{-1}s^{-1};k_{Br-}=4\times 10^{9}M^{-1}s^{-1}$$

The nitrate radical interconverts with dinitrogen pentoxide if a suitable surface is available (${NO_{3}}^{•}+NO_{2}\overset{\;}{⇌}N_{2}O_{5}$) [34]. In water N_2_O_5_ dissociates to NO_3_^−^ and NO_2_^+^; the latter pairs with a halide to form XNO_2_, which reacts with a second halide to give X_2_ [37,38]:

(5) $$X^{-}+{NO_{2}}^{+}\overset{\;}{\to }XNO_{2}\overset{\;X^{-},H^{+}\;}{\to }X_{2}+HNO_{2}$$

For chloride, Reaction (5) occurs only below pH 2 [38].

### 2.4. Speciation and Interconversion of RHS in Waters

Radical and non-radical RHS (rRHS and nrRHS) undergo well-known species and interconversion reactions in aqueous solutions. Unfortunately, rate constants are not available for iodine speciation in most cases.

Halogen atoms react rapidly and reversibly with halide ion to form the dihalogen radical anion:

(6) $$X^{•}+X^{-}\overset{\;}{⇌}{X_{2}}^{-•}$$

The equilibrium constants are large (on the order of 10^5^ M^−1^, Supplementary Table S1) and the equilibria lie far to the right in both seawater and freshwater containing typical levels of halides. When I^•^ and Br^•^ are generated, the mixed dihalogen radical anion ClX^−•^ can form, as chloride is normally predominant. The reverse of Reaction (6) preferentially gives Cl^−^ and the other halogen atom because chlorine is the most electronegative of the pair.

Kinetic modeling for seawater containing phenol in which reactions were initiated with OH^•^ indicates that the sum of all X_2_^−•^ concentrations is more than 1000-times greater than the sum of all X^•^ concentrations, and that [Br_2_^−•^] is about 2.7 times greater than [BrCl^−•^] [1].

Interconversion of halogen is possible among the rRHS. Some pertinent reactions and their equilibrium constants are given in Reactions (7) and (8):

(7) $$HOBr^{-•}+Cl^{-}\overset{\;}{⇄}BrCl^{-•}+OH^{-}\;K_{eq}=9.5$$

(8) $$HOCl^{-•}+Br^{-}\overset{\;}{⇄}BrCl^{-•}+OH^{-}\;K_{eq}=330$$

(9) $$Br_{2}{}^{-•}+Cl^{-}\overset{\;}{⇄}BrCl^{-•}+Br^{-}\;K_{eq}=5.4\times 10^{-3}$$

(10) $$BrCl^{-•}+Cl^{-}\overset{\;}{⇄}Cl2^{-•}+Br^{-}\;K_{eq}=2.75\times 10^{-8}$$

rRHS dimerize or disproportionate to give the nrRHS:

(11) $$X^{•}+X^{•}\overset{\;}{\to }X_{2}$$

(12) $$2X_{2}{}^{-•}\overset{\;}{\to }X_{2}+2X^{-}$$

(13) $$X^{•}+X_{2}{}^{-•}\overset{\;}{\to }X^{-}+X_{2}$$

Molecular halogen reacts reversibly with halide to form the trihalide ion Reaction (14). For example,

(14) $$BrCl+Cl^{-}\overset{}{⇌}BrCl_{2}{}^{-}\;K_{eq}=5.88M^{-1}$$

Pertinent to aerosol chemistry, the reactions of Cl_2_ and Br_2_ with bromide and iodide are much faster at the air-microdroplet interface than in bulk aqueous solution presumably due to differences in solvation [39]; the same is likely true for chloride but it was not included in the study.

Molecular halogen and trihalide ions hydrolyze to hypohalous acid or the hypohalite ion [40]. Some relevant reactions are:

(15) $$XCl+H_{2}O\overset{\;}{⇌}HOX+H^{+}+Cl^{-}$$

(16) $$XCl+OH^{-}\overset{\;}{⇌}HOCl+X^{-}$$

(17) $$BrCl_{2}{}^{-}+H_{2}O\overset{}{⇌}HOBr+H^{+}+2Cl^{-}\;K_{eq}=3\times 10^{-6}M^{3}$$

Reactions (15) and (16) lie far to the right and are complete within seconds.

We may consider speciation of nrRHS in different hypothetical waters (Table 1). One represents seawater (540 mM Cl^−^, 0.8 mM Br^−^, 2.3 mM carbonates, pH 8.1) [1], the other a wastewater (141 mM Cl^−^, 0.05 mM Br^−^, 11.5 mM carbonates, pH 7.0). Modeling was performed with 163 reactions using Kintecus V6.01 [41], with an OH^•^ generation rate of 1 × 10^−9^ M^−1^ s^−1^, no organic matter present, and a total simulation time of 5 or 60 min. Iodide was not included because many rate constants are unknown.

It can be seen from Table 1 that the principal X_2_ species is Br_2_ and the principal X_3_^−^ species is Br_2_Cl^−^. Among all the molecular halogen species, between 87% (seawater) and 92% (wastewater) exist as Br_2_Cl^−^ and the remainder mostly as BrCl_2_^−^. Nevertheless, the vast majority of the nrRHS are HOX/OX^−^ species, with HOBr/OBr^−^ dominating over HOCl/OCl^−^ by more than a factor of 10^3^ (wastewater) or 10^4^ (seawater). While the concentrations of all X_2_ and X_3_^−^ stay constant between 5 and 60 min, the concentrations of HOX/OX^−^ continue to increase during this interval because there is no sink for them and the starting concentrations of all reactants and the pH are held constant during the simulations. Interestingly, in seawater where chloride is at much higher concentration than in the wastewater, HOCl/OCl^−^ increases at a faster rate than HOBr/OBr^−^ between 5 and 60 min. This suggests that Br^0^ species are partially converted to Cl^0^ species over time. The most likely explanation is a series of reactions that converts HOBr to HOCl, beginning with (and probably rate-limited by) substitution of Br for Cl in HOBr:

(18) $$HOBr+Cl^{-}\overset{\;}{\to }BrCl+OH^{-}\;k=44\;M^{-1}s^{-1}$$

Following Reaction (18) would be, in sequence: (i) Reaction (14) to give BrCl_2_^−^; (ii) the reverse of Reaction (14) which gives Cl_2_ rather than Br_2_ about 5% of the time; and (iii) hydrolysis of Cl_2_ to HOCl (via Reactions (15) or (16)).

Both HOCl and HOBr readily oxidize iodide [42,43]:

(19) $$HOCl+I^{-}\overset{\;}{\to }HOI+Cl^{-}\;k=4.3\times 10^{8}\;M^{-1}s^{-1}$$

(20) $$HOBr+I^{-}\overset{k_{1}}{\to }IBr+OH^{-}\overset{k2}{\to }HOI+Br^{-}\;k_{1}=5\times 10^{9}\;M^{-1}s^{-1}\;k_{2}=6\times 10^{9}\;M^{-1}s^{-1}$$

Reactions (19) and (20) will therefore generate a lot of HOI regardless of which RHS is initially formed. In water, HOI is slowly converted to iodate (IO_3_^−^) [44]. Iodate can be an appreciable fraction of total iodine in the sea [45,46].

Since Reactions (14)–(17) are reversible, and X_2_ species are volatile, atmospheric aerosols can become depleted in bromide and iodide relative to chloride [30].

## 3. Reactions of RHS

### 3.1. Photolysis of nrRHS (X_2_, X_3_^−^, HOX)

Molecular halogens, X_2_ and X_3_^−^, all absorb at wavelengths in the solar UV and into the visible. Photolysis of X_2_ yields two X^•^ atoms [47,48] ($\Phi_{Br_{2},500nm}=0.85$ [49]; $\Phi_{IBr,500nm}=0.73$ [49]), while photolysis of X_3_^−^ yields X_2_^−•^ and X^•^ [50,51,52] ($\Phi_{Br_{3}^{-},260nm}=0.15$ [52]). However, molecular halogens are transient and their concentrations so small that photolysis is not likely an important fate mechanism.

**Scheme 3:**
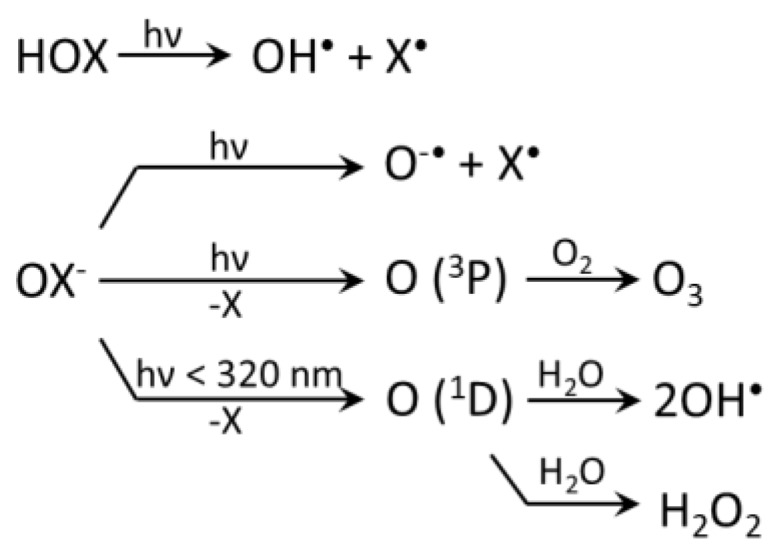
Photolysis of hypohalites.

The absorption spectra of the hypohalites partially overlap the UV solar emission spectrum, and the molar absorption of OX^−^ is greater than that of HOX. Solar UV cleaves the O-X bond homolytically or heterolytically [53,54,55,56,57] to give halogen atoms, halide ions, and a variety of ROS, including hydroxyl radical OH^•^/O^−•^ (p*K*_a_, 11.5 [58]), singlet-state atomic oxygen O(^1^D), ground-state atomic oxygen O(^3^P), ozone, and hydrogen peroxide (Scheme 3). For OCl^−^, as wavelength increases the quantum yield of homolytic cleavage decreases while that of heterolytic cleavage increases [53,59].

The absorption spectra of HOBr and HOI are red-shifted in the gas phase compared to the aqueous phase [60,61,62]. Thus, the quantum efficiency of HOBr and HOI reactions may be different in aerosols than in bulk solution due to gas-liquid interfacial effects.

### 3.2. Reactions of RHS with Inorganic Species

**Scheme 4:**
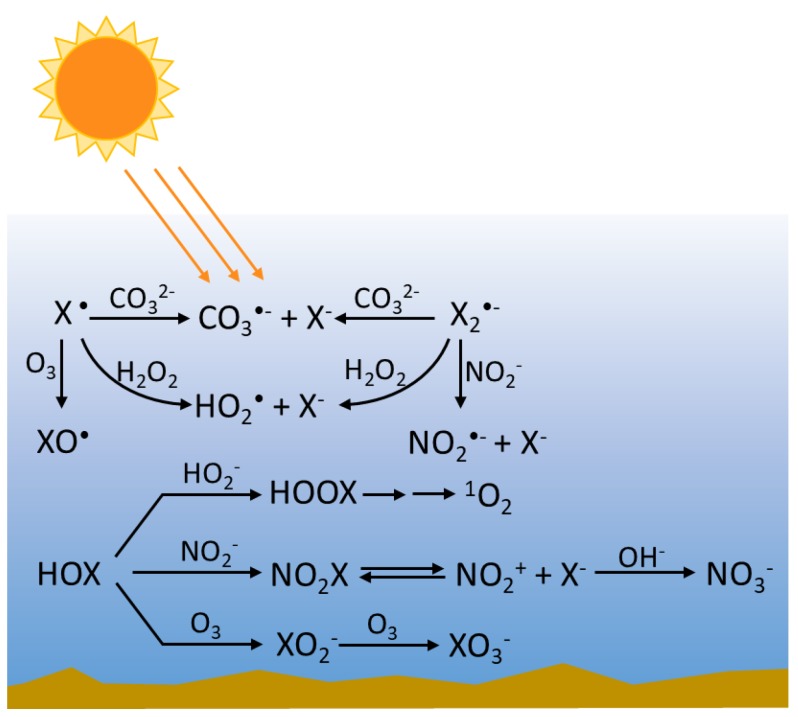
Reactions of RHS with inorganic species.

Radical and nrRHS exhibit a complex chemistry with inorganic water constituents. Potentially important scavengers include carbonates, hydrogen peroxide, nitrite, and ozone. Hydrogen peroxide is a common component of natural waters owing to biological and photochemical processes. An overview of the reactions is given in Scheme 4. As strong oxidants, RHS may also oxidize metal ions that are present at low concentrations in natural waters, such as Fe^II^, As^III^, and Mn^II^. Reactions of RHS with metal ions are covered elsewhere [63].

#### 3.2.1. Radical RHS

rRHS can be scavenged by carbonate and bicarbonate ions to form carbonate radicals, which, like rRHS, are strong oxidants of organic compounds:

(21) $$X^{•}\left(X_{2}{}^{-•}\right)+CO_{3}{}^{2-}\left(HCO_{3}{}^{-}\right)\overset{}{\to }\left(2\right)X^{-}\left(+H^{+}\right)+CO_{3}{}^{-•}\;\;k_{Cl•}=\left(0.8-5\right)\times 10^{8}M^{-1}s^{-1};k_{Br•}=\left(0.08-2.0\right)\times 10^{6}M^{-1}s^{-1}$$

Carbonates also affect RHS levels indirectly by scavenging OH^•^. Kinetic modeling shows that under OH^•^-generating conditions, addition of 2.3 mM carbonates to a solution containing 0.54 M chloride and 0.8 mM bromide steeply reduces rRHS [64]. Conversely, addition of halide ions to carbonate solutions boosts [CO_3_^−•^] [1,64] and increases the contribution of CO_3_^−•^ to transformation of phenol [1].

rRHS species oxidize H_2_O_2_ to HO_2_^•^/O_2_^−•^ Reactions (22) and (23). Rate constants are 2 × 10^9^ M^−1^ s^−1^ for Cl^•^ and 4 × 10^9^ M^−1^ s^−1^ for Br^•^, but are much smaller for X_2_^−•^ ($k_{{\mathrm{Cl}}_{2}^{\cdot -}}=1.4\times 10^{5}M^{-1}s^{-1}$; $k_{{\mathrm{Br}}_{2}^{\cdot -}}=5.0\;\times \;10^{2}M^{-1}s^{-}{}^{1}$. The products HO_2_^•^/O_2_^−•^ are not very reactive in water toward most organic compounds:

(22) $$X^{•}+H_{2}O_{2}\overset{\;}{\to }HO_{2}{}^{•}+X^{-}+H^{+}$$

(23) $$X_{2}{}^{-•}+H_{2}O_{2}\overset{\;}{\to }HO_{2}{}^{•}+2X^{-}+H^{+}$$

Nitrite reduces X_2_^−•^ to the halide and nitrite radical, NO_2_^•^:

(24) $$X_{2}{}^{-•}+NO_{2}{}^{-}\overset{}{\to }NO_{2}{}^{•}+X^{-}\;(k_{{\mathrm{Cl}}_{2}^{\cdot -}}=2.5\times 10^{8}M^{-1}s^{-1};\;k_{{\mathrm{Br}}_{2}^{\cdot -}}=2\times 10^{7}M^{-1}s^{-1})$$

Ozone reacts rapidly with Br^•^ to form XO^•^. Data are unavailable for Cl^•^ and I^•^:

(25) $$Br^{•}+O_{3}\overset{}{\to }BrO^{•}+O_{2}\;k=1.5\times 10^{8}M^{-1}s^{-1}$$

Ozone also reacts with X_2_^−•^ ($k_{{\mathrm{Cl}}_{2}^{\cdot -}}=9\times 10^{7}M^{-1}s^{-1}$). The ClO^•^ radical appears to be much less reactive than X^•^ and X_2_^−•^ toward organic compounds [65].

Since in most waters carbonates will be at millimolar concentrations, whereas H_2_O_2_, NO_2_^−^, and O_3_ will seldom exceed micromolar concentrations, scavenging of the rRHS by carbonates will usually predominate over the others. For their scavenging rates to be equal, [scavenger] = [carbonate]·*k*_carbonate_/*k*_scavenger_. Thus, for example, ozone would have to be >~1 mM for it to out-compete 1 mM CO_3_^2−^ for scavenging of Cl_2_^−•^.

#### 3.2.2. Non-Radical RHS

Hypohalites can oxidize H_2_O_2_ to give the halide and ^1^O_2_ (Reactions (26) and (27)). The highest rate constants are observed when the acidic form of one reactant is paired with the basic form of the other—namely, OX^−^ + H_2_O_2_ or HOX + HO_2_^−^. The reaction proceeds by nucleophilic attack of H_2_O_2_/HO_2_^−^ on the electrophilic halogen atom of HOX/OX^−^ to give initially H-O-O-X and then H-O-O-O-H [66], which decomposes spontaneously to ^1^O_2_ [67]. Singlet oxygen is reactive towards many compounds, but physical quenching by water severely limits this reactivity (Yang et al. manuscript in preparation).

(26) $$HOX+HO_{2}{}^{-}\overset{\;}{\to }HOOX+OH^{-}$$

(27) $$H_{2}O+HOOX\overset{\;-X^{-},-H^{+}\;}{\to }HOOOH{\overset{\;-H_{2}O\;}{\to }}^{1}O_{2}$$

Nitrite attacks hypohalites nucleophilically to generate NO_2_X Reaction (28) [68,69]. Hypochlorites are more reactive than hypobromites. At typically low NO_2_^−^ concentrations, the principal decomposition pathway of NO_2_X is reversible dissociation to X^−^ and NO_2_^+^, followed by hydrolysis of NO_2_^+^ to nitrate (Reactions (29) and (30)):

(28) $$HOX+NO_{2}{}^{-}\overset{\;}{\to }NO_{2}X+OH^{-}$$

(29) $$NO_{2}X\overset{\;}{⇄}NO_{2}{}^{+}+X^{-}$$

(30) $$NO_{2}{}^{+}+OH^{-}\overset{\;}{\to }NO_{3}{}^{-}+H^{+}$$

In acidic aerosols, it is also possible to regain the nrRHS through Reaction (5). Hypohalites react with O_3_ giving XO_2_^−^ and eventually XO_3_^−^ [25,26,70]. Bromate (BrO_3_^−^) is of concern in drinking water as a carcinogen [71]:

(31) $$XOH+O_{3}\to XO_{2}{}^{-}+O_{2}+H^{+}$$

(32) $$XO_{2}{}^{-}+O_{3}\to XO_{3}{}^{-}+O_{2}+H^{+}$$

## 4. Involvement of Halogen Species in Organic Matter Processing and Transformations of Organic Compounds

Organic matter entering natural waters is processed in part by its own photo-excitation. Photo-excitation of DOM can lead to bleaching, molecular fragmentation, and mineralization (to CO_2_). DOM can also sensitize the photolysis of dissolved compounds such as pollutants, either through direct reaction between the solute and the ^3^DOM* (via either triplet energy transfer or electron transfer [10,11]), or through reactions of the solute with secondary photoproducts of DOM such as ^1^O_2_, OH^•^, or HO_2_^•^/O_2_^−•^. Halides can affect photoexcitation and photobleaching of DOM, and give rise to RHS that can react with DOM and other organic compounds.

### 4.1. Impact of Halide Ions on Photoexcitation and Photobleaching of DOM

DOM-sensitized photolysis is an important mechanism for attenuation of organic contaminants in natural waters [10,11]. Increasing halide concentrations up to seawater levels decreased the DOM-sensitized photolysis rate of the female sex hormone, 17β-estradiol, by 90% [72]. About four fifths of the rate decrease was due to a general ionic strength effect, with the remainder to halide-specific effects, especially for bromide. The halide-specific effect was attributed to halide enhancement of DOM photobleaching, which reduced the concentration of chromophoric groups acting as sensitizers [72]. There have been a few other studies on the effects of halides on DOM-sensitized photodegradation, but they have either neglected to include ionic strength controls, or have attributed the observed effects to unrelated causes (see [72]).

Halide ions have been shown to influence the yield and lifetime of ^3^DOM*, parameters that can be measured by a sorbic acid isomerization probe method [73]. Two studies independently report substantial increases in the steady-state ^3^DOM* concentration in photolyzed water as the halide concentration increases to seawater levels [74,75]. One study [74] attributed it to a general ionic strength enhancement of ^3^DOM* lifetime by slowing intra-organic matter electron transfer, which is known to be an important decay pathway for ^3^DOM*. In the other [75], it was proposed that halides quench ^3^DOM*, but at the same time increase the rate of singlet-to-triplet intersystem crossing (^1^DOM* → ^3^DOM*). Exactly how halide affects ^3^DOM* lifetime and intersystem crossing rates remain to be resolved.

Photobleaching has important implications for the depth of the photic zone, the processing of DOM itself, and the ability of DOM to photosensitize transformations of other chemical species. The fundamental mechanisms accounting for photobleaching of DOM are not well understood, but halide ions may have an effect on rate. Using either a terrestrial DOM reference standard or an algal exudate representing seawater DOM, Mitch and co-workers [76] found that seawater levels of Cl^−^ and Br^−^ enhanced DOM photobleaching rates, independent of ionic strength. About 12% of the rate increase was attributed to the formation of RHS (from reaction with OH^•^) that target electron-rich chromophores more selectively than does OH^•^. The rest was unresolved. Studies on environmental grab samples are mixed; some report no consistent effect, others rate enhancement, and still others rate suppression with increasing salinity (see [76,77]).

### 4.2. Reactions of RHS with Organic Compounds

It is useful to summarize what is known generally about the reactions of RHS with organic compounds. The reactivities of rRHS (X^•^, X_2_^−•^) in aqueous solution have not received a great deal of attention, and rate constants are far more plentiful for X_2_^−•^ than X^•^. rRHS are commonly generated by pulse radiolysis or flash photolysis, and rate constants are calculated from the decay or growth of the UV/visible signal. As mentioned above, the major nrRHS in aqueous solution are the hypohalites, HOX and OX^−^. A sizable literature on these reactions exists due to their importance in chlorine disinfection chemistry [63,78]. To stay relevant to natural waters we will focus here mainly on initial reactions in dilute solutions.

#### 4.2.1. Radical RHS

Three major pathways for reactions of rRHS with organic compounds have been identified: H-atom abstraction from C-H groups Reaction (33); one electron removal from heteroatoms (Z = N, O, or S; Reaction (34); and addition to unsaturated bonds Reaction (35). Rate constants range from 10^4^ to 10^9^ M^−1^ s^−1^ [79,80] (http://kinetics.nist.gov/solution/):

(33) $$X^{•}\left(X_{2}{}^{-•}\right)+RH\overset{}{\to }\left(2\right)X^{-}+H^{+}+R^{•}\;\left(k=10^{7}-10^{9}M^{-1}s^{-1}{\;\mathrm{for}\;\mathrm{Cl}}^{•}{;\;10}^{3}{-10}^{6}{\;M}^{-1}s^{-1}{\;\mathrm{for}\;\mathrm{Cl}}_{2}{}^{-•}{;\;\sim 10}^{4}{\;M}^{-1}s^{-1}{\;\mathrm{for}\;\mathrm{Br}}^{•}\right)$$

(34) $$X^{•}\left(X_{2}{}^{-•}\right)+R_{n}-Z:\overset{}{\to }\left(2\right)X^{-}+R_{n}-Z^{+}{}^{•}\;\left(k={\;10}^{7}{-10}^{9}{\;M}^{-1}s^{-1}{\;\mathrm{for}\;\mathrm{Cl}}^{•}{;\;10}^{4}{-10}^{9}{\;M}^{-1}s^{-1}{\;\mathrm{for}\;\mathrm{Br}}^{•}{;\;10}^{6}{-10}^{10}{\;M}^{-1}s^{-1}{\;\mathrm{for}\;X}_{2}{}^{-•}\right)$$

(35) $$X^{•}\left(X_{2}{}^{-•}\right)+C=C\overset{}{\to }X-C-C^{•}(+X^{-})(k=10^{6}\text{–}10^{9}M^{-1}s^{-1})$$

As expected from their reduction potentials (Section 2.3), reactivity of rRHS generally follows the order: Cl^•^ > Br^•^; Cl_2_^−•^ > Br_2_^−•^; and X^•^ >> X_2_^−•^. For many organic compounds, the rate constants for reaction with Br^•^ and Cl^•^ rival those with OH^•^. While rate constants for X^•^ may exceed X_2_^−•^ by several orders of magnitude, the steady-state concentrations of the latter can exceed those of the former by several orders. Thus, both X^•^ and X_2_^−•^ must be considered. The reactivity of BrCl^−•^ with organic compounds is essentially unknown. The reduction potential of BrCl^−•^ ($E{{}^{\circ}}_{{\mathrm{BrCl}}^{\cdot -}/2{\mathrm{Cl}}^{-}}=1.85V$) lies in between that of Cl_2_^−•^ (2.30 V) and Br_2_^−•^ (1.66 V) [81], suggesting intermediate reactivity.

H-abstraction [65] seems to occur only for aliphatic C-H groups and the rate constant increases with decreasing C-H bond dissociation energy [79,80]). Molecules containing amino, hydroxyl, ether, keto, and sulfide groups preferentially undergo one-electron oxidation, as in Reaction (34).

rRHS add to the double bond alkenes reversibly Reaction (35). Rate constants for Cl_2_^−•^ and Br_2_^−•^ increase with electron-donating ability of the alkene substituents [80]. The resulting β-substituted organoradical can react with oxygen (10^8^–10^9^ M^−1^ s^−1^) to give β-halo organoperoxyl radicals (X-C-C-OO^•^) that decompose through various pathways to give such products as halohydrins, haloketones or haloaldehydes, ketones/aldehydes, epoxides, and diols.

Reactions of Br^•^, Cl_2_^−•^, and Br_2_^−•^ with simple aromatic compounds depend on substituents [80]. In general, if the substituent bears an electropositive atom with an electron pair, (e.g., -OH, OR, -NH_2_), reaction proceeds by electron transfer as in Reaction (34); whereas, if the substituent is H, alkyl, -Cl, -NO_2_, etc., the radical can add to the aromatic ring.

Mitch and co-workers [1] kinetically modeled the transformation of phenol in artificial saline media employing 180 different elementary reactions with known rate constants. They assumed that the process was initiated by photoproduction of OH^•^, and used short times to minimize involvement of nrRHS. In solutions containing just 540 mM Cl^−^ and 0.02 mM Br^−^, the contributions to phenol transformation were 74.4% by OH^•^, 21.9% by BrCl^−•^, 3.3% by Cl_2_^−•^, and 0.4% by Br_2_^−•^. In simulated seawater that included 2.3 mM carbonates, they were 52.6% by CO_3_^−•^, 6% by OH^•^, 21.5% by ClBr^−•^, 0.1% by Cl_2_^−•^, and 19.7% by Br_2_^−•^. The lessons to be learned from this with respect to phenol transformation are that, (a) carbonates divert oxidative power away from OH^•^ toward CO_3_^−•^ and X_2_^−•^; (b) X^•^ seems to play no significant role; and (c) BrCl^−•^, is an important rRHS.

#### 4.2.2. Non-Radical RHS

Hypohalites react with organic compounds by electrophilic substitution, electrophilic addition, or oxidation. Known apparent second-order rate constants for HOCl reactions with organic compounds range widely from 10^−2^ to 10^7^ M^−1^ s^−1^ [63]. The most reactive functional groups are amino, keto/aldehyde, phenolic, and low-valent sulfur.

The neutral form of amines reacts rapidly with HOCl (primary > secondary >> tertiary) to form chloramines Reaction (36). α-Amino acid groups undergo further decarboxylation and deamination reactions [63]:

(36) $$R-NH_{2}+HOCl\overset{\;}{\to }R-NHCl+H_{2}O$$

α-Amino acid groups undergo further decarboxylation and deamination reactions [63]. Aromatic compounds react with HOX by electrophilic substitution of halogen. HOBr and HOI are more reactive than HOCl [42,82]. Ring substituents increase the rate constant in the approximate order, R– < RO– < HO– < (HO–)*_n_* > _1_. Phenols give *o-* and *p*-X substituted products. The phenoxide ion is ~10^5^-times more reactive than the free phenol, and reactivity correlates with electron donor character of the substituents. *Ortho*- and *para*-substituted dihydroxyaromatics undergo oxidation to the corresponding quinone [83].

Above pH ~ 5 ketones and aldehydes are halogenated by electrophilic substitution at the α carbon Reaction (37), an important reaction in disinfection chemistry because it leads to hazardous byproducts:

(37) $$R-C\left(=O\right)-CH_{3}\overset{\;OH^{-}\;}{⇄}R-C\left(-O^{-}\right)=CH_{2}\overset{\;HOX\;}{\to }R-C\left(=O\right)-CH_{2}Cl$$

Alkenes are attacked electrophilically by the halogen atom of HOX at the least-substituted end of the double bond to form the halohydrin Reaction (38).

(38) $$C=C+HOX\overset{\;}{\to }X-C-C-OH$$

The halohydrin can undergo internal or solvolytic elimination of halide to form, respectively, the epoxide or the α,β-dihydroxy compound.

Hypohalites can also oxidize some functional groups—for example, primary and secondary alcohols to aldehydes and ketones, respectively; aldehydes to carboxylic acids; and sulfides to sulfones, sulfoxides, or sulfonic acids [78]. Some of those reactions may go through halogenated intermediates via electrophilic pathways.

### 4.3. Photo-Initiated Incorporation of Halogen into Organic Compounds under Natural Conditions

Given that RHS are photochemically produced in natural waters, the question arises as to whether this could lead to incorporation of halogen into natural compounds and water contaminants. Of the nearly five thousand natural organohalogen compounds identified to date [84], only a few have been assigned an abiotic origin. It is important to understand abiotic halogenation pathways because organohalogen compounds are known to play important roles in climate warming, ozone depletion, and toxicity. In addition, abiotic halogenation may play a role in pollutant fate.

#### 4.3.1. Incorporation of Halogen into Simple, Defined Molecules

A number of volatile gases of importance in stratospheric ozone chemistry and climate warming are thought to originate from abiotic reactions in the oceans. Table 2 lists examples of these compounds and their origins. Moreover, sunlight illumination of natural and artificial saline samples has been observed to halogenate specific organic probe compounds. Table 2 also lists these compounds, which include natural compounds, lignin-like model compounds representing NOM, and pollutants. The mechanism of halogenation is unambiguously established in few of these cases.

A noteworthy example of abiotic halogenation of a natural compound with potentially important ramifications for animal and human exposure was recently reported by Kumar et al. [27]. They found that ozonation of seawater samples in the dark stimulated polyhalogenation (X = Br, Cl) of 1,1-dimethyl-2,2′-bipyrrole and 1′-methyl-1,2′-bipyrrole. The polyhalogenated derivatives of these two bipyrroles are widely distributed among oceanic sealife and found in air samples and human breastmilk, but a satisfactory biological explanation for their existence has been elusive. Under laboratory ozonation conditions, bromination predominated over chlorination and there was no detectable iodine incorporation. Kumar et al. [27] proposed that tropospheric ozone exchanging with seawater generates HOCl and HOBr (see Reaction (3)), which halogenates the bipyrroles. The presence of chlorine RHS is unexpected because, at seawater halide levels, bromide reacts ~130 times faster than chloride with O_3_ (see Section 2.2). However, since rather high ozone concentrations were employed (0.2–2.2 mmol/L), it is possible that bromide became depleted in solution. Alternative explanations for the chlorinated products are conversion of some bromine RHS to chlorine RHS, or nucleophilic displacement of bromide by chloride on intermediate products. Interestingly, in kinetic modeling of hydroxyl radical-generating systems in water containing 540 mM NaCl and 0.02 mM NaBr, 3.3% of phenol degradation was due to reaction with Cl_2_^−^·, which could only come from oxidation of chloride [1]. The Cl_2_^−•^ may originate from chloride displacement of hydroxide from the reversibly-formed species, ClOH^−•^:

(39) $$Cl^{-}+OH^{•}\overset{k=4.3x10^{9}M^{-1}s^{-1}}{\underset{k=6.1x10^{9}M^{-1}s^{-1}}{⇄}}ClOH^{-•}\overset{\;Cl^{-};k=1x10^{5}M^{-1}s^{-1}\;}{\to }Cl_{2}{}^{-•}+OH^{-}$$

Or it could come from chloride displacement of bromide in BrCl^−•^ Reaction (10; *k* = 1.1 × 10^2^ M^−1^ s^−1^).

Mitch and co-workers [1] investigated halogen incorporation into phenol both in artificial seawater and wastewater concentrate (141 mM NaCl, 0.05 mM NaBr, and 11.5 mM carbonates at pH 7.0) spiked with H_2_O_2_ and irradiated with UV for 35 min. Phenol can be considered a model compound for terrestrial DOM and some pollutants. Both chloro- and bromophenols were produced, with bromophenols constituting the majority of products. However, the total yields based on initial phenol were only 0.52% in seawater and 0.03% in wastewater concentrate. The yields were unaffected by eliminating the carbonate component, despite carbonate’s ability to scavenge rRHS Reaction (19). While not established by these results, it is more likely that phenol was halogenated by nrRHS, given the greater reactivity of nrRHS than rRHS toward phenolic compounds (Section 4.2).

#### 4.3.2. Incorporation of Halogen into Bulk DOM

Recent studies [94,95] show convincingly that bulk natural organic matter is photo-halogenated under natural or simulated natural conditions. In the study by Mitch, Dodd, and co-workers [94], organo-Br and organo-I were quantified by solid-phase extraction and silver-form cation exchange filtration to remove the high background of halide ions, followed by non-specific quantification of Br and I by inductively-coupled plasma mass spectrometry (ICP-MS) (the method was insensitive for Cl). In the study by Hao et al. [95], the organohalogen compounds were identified at the formula level by ultra-high resolution electrospray ionization Fourier transform ion cyclotron resonance mass spectrometry (ESI-FT ICR MS).

Native organobromine and organoiodine compounds were found in a variety of seawater samples at concentrations ranging (3.2–6.4) × 10^−4^ mol Br/mol C and (1.1–3.8) × 10^−4^ mol I/mol C (or 19–160 nmol Br L^−1^ and 6–36 nmol I L^−1^) [94,95], and diminishing with ocean depth [94]. Simulated and natural solar irradiation of terrestrial NOM spiked to artificial and natural seawaters led to halogenation that increased with light fluence [94]. With added NaI, iodination increased at the expense of bromination [94]. Addition of the probe, 3,5-dimethyl-1*H*-pyrazole (DMP), to irradiated natural seawater samples generated 4-Br and 4-I DMP. Since rRHS oxidize rather than halogenate DMP, this result verifies production of nrRHS in these systems [94] and points to nrRHS as the most likely source of halogenated DOM.

Control experiments indicated that some of the native and photo-generated organobromine and organoiodine compounds are photolabile [94,95]. This indicates that the prevailing levels of organohalogen found in environmental samples likely reflect a balance between formation and decomposition. Experiments in artificial seawater showed that chloride ion stimulates organobromine production [94,95]. This implies that chloride facilitates oxidation of bromide. If ^3^DOM* is the active oxidant species, one may postulate a mechanism involving the formation and subsequent decay of a ternary exciplex, as previously discussed (Section 2.1):

(40) DOM*3+Br−+Cl−→3[DOM−−−−Br•−−−Cl−]→ DOM−•3+BrCl−•

The ESI-FT ICR-MS study provided a wealth of information on the types of reactions that occur [95]. Most native and photo-produced organohalogen compounds were mono- or di-Br or I molecules (a few contained Cl) of 250–700 Daltons in size, and there was considerable overlap among the natively-present and photo-produced compounds. Some products could be attributed to simple H-for-X substitution or X-addition reactions, but most were the result of multiple processes, often accompanied by photooxidation. Most brominated compounds fell in regions of the van Krevelin diagram indicating derivation from unsaturated aliphatic compounds and saturated fatty acids and carbohydrates, while smaller numbers were derived from polycyclic aromatic and polyphenol moieties. Most iodo compounds appeared to be derived mainly from lignin- or tannic-like structures.

In summary, the results suggest that sunlight-driven reactions of RHS with DOM play an important role in bromine and iodine geochemical cycling in marine environments. It has been estimated [94] that photochemical halogenation of terrestrial DOM in estuaries could generate 30 Gg of organobromine and 70 Gg of organoiodine annually worldwide. Those values do not even include RHS-driven halogenation of marine DOM in the open ocean.

## 5. Impacts of Halides on Water Treatment Processes

Photo-driven AOPs using oxygen, ozone, and peroxides as bulk oxidants are frequently used to destroy pollutants in drinking water and wastewaters. Semiconductor materials are often used as photocatalysts. While earlier work employed UV light, recent emphasis has been on reactions that are viable in the visible or solar spectral regions to reduce energy costs. Wastewaters such as landfill leachates, production waters, industrial wastewaters, and reverse osmosis brines intended for reuse or safe disposal often contain high levels of halide ions. Application of AOPs for treating salty waters is challenging due, among other things, to the conversion of ROS to RHS, which can affect the efficiency of organic compound degradation and generate unwanted halogenated byproducts. It should be noted that solutions irradiated with UV wavelengths below the absorption edges of halide ions (~260 nm) may generate rRHS from direct photolysis of halides Reaction (41):

(41) $$X^{-}\overset{\;UV\;}{⇄}(X^{•},e^{-})\overset{\;H^{+}(H_{2}O)\;}{\to }X^{•}+H^{•}(+OH^{-})$$

This reaction proceeds through a reversible halogen atom-electron solvent cage complex that produces free halogen and hydrogen atoms upon reaction with water [96,97]. The hydrogen atoms are normally rapidly scavenged by O_2_ to produce HO_2_^•^/O_2_^−•^. One study [97] reports that Reaction (41) can be driven in the bay of a diode array spectrophotometer (!), and cautions about the potential for analytical interference.

### 5.1. Hydroxyl Radical-Based AOPs

Numerous AOPs generate OH^•^, including H_2_O_2_/UV, Fenton reactions, and heterogeneous photocatalysis (e.g., TiO_2_), among others. There are several reports of decreased rates and organohalogen formation when OH^•^-based AOPs were conducted in the presence of elevated halide concentrations. A few examples are given. One study involving a Fenton-based AOP to destroy dyes indicated that dye removal decreased while total halogenated organic compounds (AOX) increased when 57 mM Cl^−^ was present at pH 2.8 and 1 [98]. Another observed auto-inhibition of 1,2-dibromoethane oxidation in a (dark) Fenton-based AOP due to the generation of bromide ions during the reaction that scavenged OH^•^ [99]. Machulek observed that chloride inhibited mineralization of organic compounds by the photo-Fenton reaction, both in a synthetic phenol wastewater and an extract of gasoline [100]. In Fenton reactions at pH 2.8, the impact of chloride scavenging on organic compound transformation rate was noticeable above 0.01 M Cl^−^ [21].

Kinetic modeling of phenol oxidation by an OH^•^-generating reaction (H_2_O_2_/UV) in phosphate-buffered water containing 0.8 mM NaBr showed that OH^•^ accounted for most (74%) of phenol transformation, Br_2_^−•^ for 24%, and Br^•^ for 0.8% [1]. In a synthetic wastewater (141 mM chloride, 0.05 mM bromide, 11.5 mM carbonates) at pH 7, OH^•^ was still the most important radical (67%), followed by CO_3_^−•^ (31%), BrCl^−•^ (2.1%), and Br_2_^−•^ (0.3%).

It has been proposed that halide ions can be oxidized on the surfaces of semiconductor photocatalysts such as TiO_2_, either by surface-associated hydroxyl radicals or valence band holes [101,102]. It is known that chloride forms an inner-sphere complex with Ti at the surface [103].

### 5.2. The UV/Chlorine AOP

The UV photolysis of HOCl has been proposed as an alternative AOP. The photolysis of HOCl/OCl^−^ at 254 nm yields OH^•^ and Cl^•^ (Section 3.1; Scheme 3). Some OH^•^ and Cl^•^ are scavenged by HOCl/OCl^−^, however, to give ClO^•^, which is a less-reactive radical towards organics [65,104]:

(42) $$OH^{•}/Cl^{•}+HOCl/OCl^{-}\overset{\;}{\to }ClO^{•}$$

In bromide-containing waters, HOCl/OCl^−^ rapidly converts to HOBr/OBr^−^ [105]. Photolysis of HOBr/OBr^−^ generates OH^•^ and Br^•^ (Scheme 3) [106]. HOBr is also known to oxidize HOI to IO_3_^−^. [107,108] When HOCl is in excess, the oxidation of I^−^ to IO_3_^−^ is catalyzed by Br^−^. Obviously, the use of UV/chlorine AOP has the potential to generate high levels of halogenated byproducts.

### 5.3. Sulfate Radical-Based AOPs

Sulfate radical (SO_4_^−•^)-based AOPs are attractive alternatives to hydroxyl radical-based AOPs. The sulfate radical is nearly as reactive toward organic compounds as hydroxyl. UV/peroxydisulfate (S_2_O_8_^2−^) has a higher quantum yield of SO_4_^−•^ from S_2_O_8_^2−^ (1.4) at 254 nm [109] than does OH^•^ from H_2_O_2_ (1.0) [110]. The photolysis of peroxymonosulfate at 254 nm generates OH^•^ and SO_4_^−•^ simultaneously [111]. Sulfate radical can also be generated from peroxysulfates by various non-photolytic means, as well. Sulfate radicals convert to hydroxyl radicals in water above pH 7.

Sulfate radical reacts directly and rapidly with the halide ions:

(43) $$SO_{4}{}^{-•}+X^{-}\overset{\;}{\to }SO_{4}{{}^{2}}^{-}+X^{•}$$

While oxidation of Cl^−^ by OH^•^ is important only in acidic solution, oxidation of Cl^−^ by SO_4_^−•^ Reaction (43) is pH-independent and therefore impacts water treatment over a much broader pH range. Several studies have shown that halide ions can strongly affect SO_4_^−•^-based oxidation rates [64] and lead to halogenated byproducts [112].

Experiment and kinetic modeling show that SO_4_^−•^-based processes are more strongly affected by halides than are OH^•^-based processes [64]. In the presence of halides and carbonates, the steady-state concentrations of X_2_^−•^ and CO_3_^−•^ are much higher than those of SO_4_^−•^ and OH^•^ [80,113]. This, combined with the fact that X_2_^−•^ and CO_3_^−•^ are typically less reactive and more selective toward organic compounds than SO_4_^−•^ and OH^•^, means that oxidation efficiency can be significantly impacted [80,113]. However, the impact depends on molecular structure. Benzoic acid transformation by UV/S_2_O_8_^2−^ was strongly suppressed in 0.54 M chloride solution compared to phosphate-buffered water, while cyclohex-3-ene carboxylic acid was hardly affected at all [64]. This is because the major rRHS that formed, Cl_2_^−•^, is poorly reactive toward benzoic acid, but highly reactive toward the double bond in cyclohex-3-ene carboxylic acid [64]. A similar reason was offered to explain the effects of halides and carbonates on the UV/S_2_O_8_^2−^ reactivity of different pharmaceuticals in reverse-osmosis brine compared to water—namely, that X_2_^−•^ and CO_3_^−•^ were more reactive toward some than others [114].

**Scheme 5:**
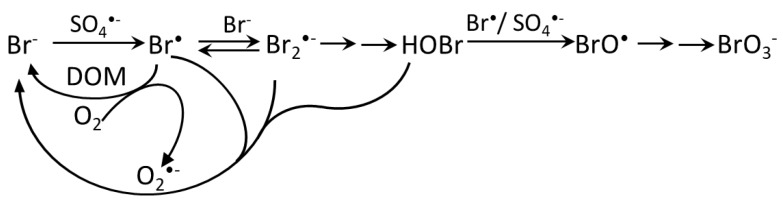
The mechanism of BrO_3_^−^ formation by SO_4_^−•^.

Sulfate radical AOPs can yield bromate (BrO_3_^−^) as a final product under some conditions (Scheme 5) [115,116]. Bromate is a suspected human carcinogen with a drinking water standard of 10 μg/L as set by U.S. EPA and the World Health Organization [117]. Both experiment and modeling indicate that HOBr/OBr^−^ is a required intermediate in the production of bromate (Scheme 5) [115,116]. The yield of bromate is pH-dependent, as HOBr is about 2 orders of magnitude less reactive than OBr^−^ toward Br^•^ [116,118]. Organic solutes, DOM, and generated superoxide can scavenge Br^•^, Br_2_^−•^, and HOBr. This has the effect of significantly reducing or eliminating bromate formation, as well as recycling bromine back into the bromide form [116].

**Scheme 6:**
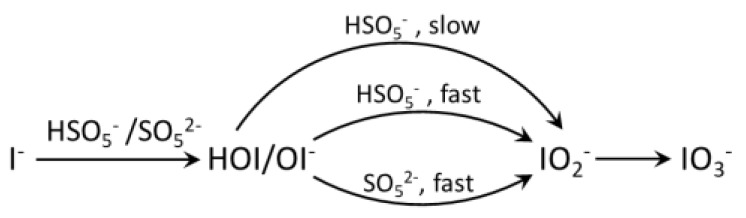
The mechanism of IO_3_^−^ formation by peroxymonosulfate oxidation of iodide.

Halides can also react directly with peroxymonosulfate. The bimolecular rate constant follows the order I^−^ > Br^−^ > Cl^−^ [119]. The evidence is consistent with nucleophilic attack of halide on the peroxy group. The product HOX (except HOCl) is further oxidized by peroxymonosulfate [120]. Oxidation of HOI to IO_2_^−^ is strongly pH dependent due to speciation effects [121] (Scheme 6.). The reaction of IO_2_^−^ to IO_3_^−^ is very fast. The oxidation of HOBr to BrO_3_^−^ is much slower [120]:

(44) $${}^{-}O_{3}SOOH+X^{-}\overset{\;}{\to }HOX+SO_{4}{{}^{2}}^{-}\;k_{Cl}=2.1\times 10^{-3}M^{-1}s^{-1};k_{Br}=0.7M^{-1}s^{-1};k_{I}=1.1\times 10^{3}M^{-1}s^{-1}$$

In the presence of iodide, peroxymonosulfate reactions can also lead to incorporation of iodine into DOM and form byproducts of concern derived from DOM, namely, iodoform (CHI_3_) and iodoacetic acid [121].

## 6. Concluding Remarks

Halogen plays an important and colorful role in environmental photochemical processes in natural waters and in chemical reactions taking place during photochemical water purification. In the environment, halides can be oxidized to rRHS (X^•^, X_2_^−•^) principally through DOM-sensitized photolysis and reactions with ROS of photochemical origin, especially hydroxyl radical, but also ozone and nitrate radical in atmospheric aerosols. Much more work needs to be done to establish the mechanism and importance of DOM-sensitized photolysis of halide ions with respect to generation of RHS. The nature of the chromophoric groups and the quantum yields of initial RHS products as a function of DOM type need to be established. It is noteworthy that chloride enhances bromination and further work is needed to establish whether the cause is formation of a ternary exciplex like the one in Scheme 2. DOM photosensitization of RHS formation is diminished with photobleaching of DOM, a process that itself is affected by halide ions.

rRHS dimerize or disproportionate to give nrRHS, chiefly the hypohalites (HOX) interconverting with smaller amounts of molecular halogen species, X_2_ and X_3_^−^. nrRHS can photolyze to regenerate halogen atoms and produce hydroxyl radical, ozone, or hydrogen peroxide, depending on pH and wavelength. Hypohalites can react with hydrogen peroxide, nitrite, and ozone to give singlet oxygen, nitrate, and oxyhalide anions, respectively—products that oxidize organic compounds less efficiently than the hypohalites. Rate constants are lacking for many speciation reactions and reactions of RHS with other photo-generated species, especially in the case of I. Usually the most important inorganic scavenger of rRHS will be the carbonates.

Halide ions at relatively high concentrations can apparently increase the steady-state concentration of excited triplet-state dissolved organic matter (^3^DOM*). Exactly how this happens is not entirely clear and deserves further research; one study attributed it to a general ionic strength effect, while another to an increase in the rate of singlet-to-triplet intersystem crossing. Some studies report that high halide concentration accelerates photobleaching of DOM. The mechanism has not been established with confidence. An increase in the steady-state ^3^DOM* concentration could lead to a loss of chromophoric groups through intra-DOM reactions, or an increase in the generation of RHS that can oxidize DOM.

Reactions of X^•^, X_2_^−•^, and HOX with organic compounds in water have been characterized, although rate constants are sparse for iodine compounds and for X^•^ relative to X_2_^−•^. Rate constants for the mixed species, BrCl^−•^, are unavailable. Depending on structure, rRHS can react by H-atom abstraction, one-electron oxidation, and addition to double bonds and aromatic rings. The addition reactions can lead to incorporation of halogen into the products. Hypohalites react principally by non-radical electrophilic reactions, including halogen incorporation into amines, ketones, alkenes, and aromatic rings. Incorporation of halogen seems to be more likely with nrRHS than rRHS. Hypohalites can also oxidize alcohols, aldehydes, and sulfides without halogen incorporation. Limited interconversion of halogen within and between rRHS and nrRHS is possible, and can lead to incorporation of all halogens into organic molecules, regardless of which RHS species are generated initially. Some naturally-occurring halogenated compounds are known to form abiotically, including ozone-depleting gases. Many of these are thought to have a photolytic origin. Evidence has appeared for abiotic incorporation of halogen into water contaminants and model compounds representing natural organic matter initiated by photochemical processes. Evidence has also appeared for the photochemical incorporation of halogen into natural compounds creating products toxic to oceanic sealife. More examples of natural abiotic incorporation of halogen atoms into natural compounds and water contaminants are likely to appear in the future.

Recent studies show that DOM from both oceanic and terrestrial sources is halogenated under simulated or natural conditions of irradiation. The mechanisms of halogen incorporation have not been identified precisely. Likewise, the scope of such reactions and the effects of water chemistry are as yet poorly characterized. Complicating matters is the finding that photo generation and decomposition of halogenated DOM seem to be taking place simultaneously. The results so far indicate that sunlight-driven oxidation by RHS and halogenation reactions may play important roles in halogen geochemical cycling in marine and estuarine environments, especially in regard to bromine and iodine. It is possible that the presence of natural halogenated compounds has contributed to the evolution of enzymatic dehalogenation pathways of halogenated molecules.

Oxidation and halogen incorporation are of demonstrated importance in AOPs for salty water treatment that use light. Rates can be markedly slowed (or sometimes accelerated) and undesirable byproducts can be formed. There is much to be learned about the influence of halide salts. It is noteworthy that halides can be photolyzed by UV below 260 nm. Whether halides are oxidized on photocatalyst surfaces is largely an open question. The rate constants of RHS with many organic compounds are unknown, but important for evaluating the contribution of RHS to organic compound degradation. The yields of halogenated byproducts depend strongly on the parent compound and the solution conditions. An insufficient database exists to predict precisely where rRHS will attack in a complex molecule. The halogenated byproducts may be of greater toxicity than the original contaminants. Although many studies reported the appearance of halogenated products, quantitative yields are often not reported [101,104,122]. For these reasons the impact of halides on toxicity of the treated waters should routinely accompany investigations, and such information should be used to judge the suitability of the AOP.

## Figures and Tables

**Table 1:** Simulated speciation of nrRHS in different waters. Molar ratio relative to Cl_2_ after 5 min except where noted.

RHS/Cl_2_	Br_2_	BrCl	Cl_3_^−^	BrCl_2_^−^	Br_2_Cl^−^	Br_3_^−^	HOBr/OBr^−^	HOCl/OCl^−^
Wastewater	4.01 × 10^3^	2.27	0.0257	33.5	417	3.5	0.95 × 10^9^ (1.74 × 10^10^) *	2.57 × 10^5^ (5.92 × 10^5^) *
Seawater	1.04 × 10^4^	24.7	0.0982	533	3800	145	6.42 × 10^9^ (7.08 × 10^9^) *	1.79 × 10^5^ (6.2 × 10^5^) *

* After 60 min.

**Table 2:** Some examples of halogenation reactions of specific organic compounds in illuminated salty water systems.

Compound	Proposed Origin	References
CH_3_Cl	(a) nucleophilic displacement by chloride on CH_3_I and/or CH_3_Br in seawater; (b) is produced on irradiation of lignin-like DOM model compounds (4-methoxy-1-naphthol; syringic acid; 2-methoxyphenol; 3,4,5-trimethoxy benzoic acid; and2-methoxyhydroquinone) in chloride solution	(a) [85] (b) [86]
CH_3_I	formed after simulated solar irradiation of filtered seawater; production was enhanced when samples were degassed or iodide was added; proposed origin is recombination of CH_3_^•^ and I^•^ radicals.	[87]
CH_2_I_2_, CHI_3_, and CHI_2_Cl	formed by reactions of DOM with HOI generated via oxidation of I^−^ by O_3_	[88]
CH_2_ICl	photolysis product of CH_2_I_2_ in seawater	[89]
Cl-CH_2_CH(OH)CH_2_OH and Br-CH_2_CH(OH)CH_2_OH	CH_2_=CHCH_2_OH reaction with reactive halogen species	[90]
3-Cl and 3,3-diCl bisphenol A	solar irradiation of bisphenol A in coastal seawater and saline solution containing 0.13–0.66 mM Fe(III) and fulvic acid; Cl_2_^−•^ was detected by its absorption spectrum, and OH^•^ as its DMPO adduct by EPR spectroscopy; proposed source of halogen radicals: Fe^III^Cl^−^ → Fe^II^ + Cl^•^ or Fe^III^OH^−^ → Fe^II^ + OH^•^, followed by OH^•^ + Cl^−^ → Cl^•^.	[91]
5-bromo-and 3,5-dibromosalicylic acids	solar irradiation of salicylic acid in artificial seawater and brackish lagoon water	[92]
mixed poly-brominated/chlorinated bipyrroles	irradiation of 1,1-dimethyl-2,2′-bipyrrole and 1’-methyl-1,2’bipyrrole in ozonated seawater; proposed oxidation of Br^−^ and I^−^ by O_3_ to form HOX/X_2_.	[27]
halogenated dicarboxylic acids	isolated from arctic aerosols; unclear whether transformations occurred in the liquid phase	[93]

## Supplementary Materials

The following are available online. Table S1: Rate constants for relevant reactions of halides and reactive halogen species.
