# Supplementary material for: Participation of the Halogens in Photochemical Reactions in Natural and Treated Waters
Source: Molecules. 2017 Oct 13;22(10):1684. doi: 10.3390/molecules22101684 (PMC6151492; doi:10.3390/molecules22101684)
Supplement: Supplementary file 1 [file molecules-22-01684-s001.pdf]

SUPPLEMENTAL SECTION

PARTICIPATION OF THE HALIDES IN PHOTOCHEMICAL REACTIONS IN NATURAL WATERS AND TREATED WATERS

Yi Yang and Joseph J. Pignatello*

Department of Environmental Sciences, The Connecticut Agricultural Experiment Station, 123 Huntington St., P.O. Box 1106, New Haven, Connecticut, U.S.A

Table S1. Rate constants for relevant reactions of halides and reactive halogen species.

**No.**	**Reaction**	**Rate Constant**	**Reference**
Chloride reaction			
1*	$Cl^{-} + {}^{\bullet}OH \rightarrow ClOH^{\bullet-}$	4.3×10^{9} M^{-1}s^{-1}	[1]
2	$Cl^{-} + SO_4^{\bullet-} \rightarrow Cl^{\bullet} + SO_4^{2-}$	3.0×10^{8} M^{-1} s^{-1}	[2]
3	$Cl^{\bullet} + SO_4^{2-} \rightarrow Cl^{-} + SO_4^{\bullet-}$	2.5×10^{8} M^{-1} s^{-1}	[2]
4	$Cl^{-} + NO_3^{\bullet} \rightarrow Cl^{\bullet} + NO_3^{-}$	3.5×10^{8} M^{-1} s^{-1}	[3]
5	$Cl^{\bullet} + NO_3^{-} \rightarrow Cl^{-} + NO_3^{\bullet}$	1.0×10^{8} M^{-1} s^{-1}	[3]
6*	$ClOH^{\bullet-} \rightarrow {}^{\bullet}OH + Cl^{-}$	6.1×10^{9} M^{-1}s^{-1}	[1]
7*	$ClOH^{\bullet-} + H^{+} \rightarrow Cl^{\bullet} + H_2O$	2.1×10^{10} M^{-1}s^{-1}	[1]
8*	$ClOH^{\bullet-} + Cl^{-} \rightarrow Cl_2^{\bullet-} + OH^{-}$	1.0×10^{4} M^{-1}s^{-1}	[4]
9*	$Cl^{\bullet} + H_2O \rightarrow ClOH^{\bullet-} + H^{+}$	2.5×10^{5} M^{-1}s^{-1}	[1]
10*	$Cl^{\bullet} + OH^{-} \rightarrow ClOH^{\bullet-}$	1.8×10^{10} M^{-1}s^{-1}	[5]
11*	$Cl^{\bullet} + H_2O_2 \rightarrow HO_2^{\bullet} + Cl^{-} + H^{+}$	2.0×10^{9} M^{-1}s^{-1}	[6]
12*	$Cl^{\bullet} + Cl^{-} \rightarrow Cl_2^{\bullet-}$	8.5×10^{9} M^{-1}s^{-1}	[6]
13*	$Cl^{\bullet} + Cl^{\bullet} \rightarrow Cl_2$	8.8×10^{7} M^{-1}s^{-1}	[7]

14*	$Cl^{\bullet} + HOCl \rightarrow ClO^{\bullet} + H^{+} + Cl^{-}$	3.0×10^{9} M^{-1}s^{-1}	[5]
15*	$Cl^{\bullet} + OCl^{-} \rightarrow ClO^{\bullet} + Cl^{-}$	8.3×10^{9} M^{-1}s^{-1}	[5]
16*	$Cl_2^{\bullet-} \rightarrow Cl^{\bullet} + Cl^{-}$	6.0×10^{4} M^{-1}s^{-1}	[6]
17*	$Cl_2^{\bullet-} + {}^{\bullet}OH \rightarrow HOCl + Cl^{-}$	1.0×10^{9} M^{-1}s^{-1}	[8]
18*	$Cl_2^{\bullet-} + Cl_2^{\bullet-} \rightarrow Cl_2 + 2Cl^{-}$	9.0×10^{8} M^{-1}s^{-1}	[6]
19*	$Cl_2^{\bullet-} + Cl^{\bullet} \rightarrow Cl_2 + Cl^{-}$	2.1×10^{9} M^{-1}s^{-1}	[6]
20*	$Cl_2^{\bullet-} + H_2O_2 \rightarrow HO_2^{\bullet} + 2Cl^{-} + H^{+}$	1.4×10^{5} M^{-1}s^{-1}	[9]
21*	$Cl_2^{\bullet-} + HO_2^{\bullet} \rightarrow O_2 + 2Cl^{-} + H^{+}$	3.0×10^{9} M^{-1}s^{-1}	[9]
22*	$Cl_2^{\bullet-} + O_2^{\bullet-} \rightarrow O_2 + 2Cl^{-}$	2.0×10^{9} M^{-1}s^{-1}	[9]
23*	$Cl_2^{\bullet-} + H_2O \rightarrow Cl^{-} + HClOH^{\bullet}$	1.3×10^{3} M^{-1}s^{-1}	[10]
24*	$Cl_2^{\bullet-} + OH^{-} \rightarrow Cl^{-} + ClOH^{\bullet-}$	4.5×10^{7} M^{-1}s^{-1}	[4]
25	$Cl_2^{\bullet-} + NO_3^{\bullet} \rightarrow Cl_2 + NO_3^{-}$	1.0×10^{9} M^{-1} s^{-1}	[3]
26	$Cl_2^{\bullet-} + NO_2^{\bullet} \rightarrow Cl_2 + NO_2^{-} or\ 2Cl^{-} + NO_2^{+}$	≦1.0×10^{9} M^{-1} s^{-1}	[3]
27*	$HClOH^{\bullet} \rightarrow ClOH^{\bullet-} + H^{+}$	1.0×10^{8} M^{-1}s^{-1}	[10]
28*	$HClOH^{\bullet} \rightarrow Cl^{\bullet} + H_2O$	1.0×10^{2} M^{-1}s^{-1}	[10]
29*	$HClOH^{\bullet} + Cl^{-} \rightarrow Cl_2^{\bullet-} + H_2O$	5.0×10^{9} M^{-1}s^{-1}	[10]
30*	$Cl_2 + Cl^{-} \rightarrow Cl_3^{-}$	2.0×10^{4} M^{-1}s^{-1}	[11]
31*	$Cl_3^{-} \rightarrow Cl_2 + Cl^{-}$	1.1×10^{5} M^{-1}s^{-1}	[11]
32*	$Cl_3^{-} + HO_2^{\bullet} \rightarrow Cl_2^{\bullet-} + HCl + O_2$	1.0×10^{9} M^{-1}s^{-1}	[12]
33*	$Cl_3^{-} + O_2^{\bullet-} \rightarrow Cl_2^{\bullet-} + Cl^{-} + O_2$	3.8×10^{9} M^{-1}s^{-1}	[13]

34*	$Cl_2 + \mathrm{H_2O} \rightarrow Cl^- + HOCl + H^+$	15 $M^{-1}s^{-1}$	[14]
35*	$Cl_2 + \mathrm{H_2O_2} \rightarrow O_2 + 2HCl$	1.3×10^4 $M^{-1}s^{-1}$	[13]
36*	$Cl_2 + O_2^{\bullet-} \rightarrow O_2 + Cl_2^{\bullet-}$	1.0×10^9 $M^{-1}s^{-1}$	[13]
37*	$Cl_2 + HO_2^{\bullet} \rightarrow H^+ + O_2 + Cl_2^{\bullet-}$	1.0×10^9 $M^{-1}s^{-1}$	[12]
38*	$ClO_2^{\bullet} + {}^{\bullet}OH \rightarrow ClO_3^{-} + H^+$	4×10^9 $M^{-1}s^{-1}$	[15]
39	$ClO_2^{\bullet} + O_3 \rightarrow ClO_3^{-} + O_2$	6×10^8 $M^{-1}s^{-1}$	[15]
40*	$HOCl \rightleftarrows ClO^- + H^+$	$K_{eq} = 2.82\times10^{-8}$	[16]
41*	$HOCl + H_2O_2 \rightarrow HCl + H_2O + O_2$	1.1×10^4 $M^{-1}s^{-1}$	[17]
42*	$OCl^- + H_2O_2 \rightarrow Cl^- + H_2O + O_2$	1.7×10^5 $M^{-1}s^{-1}$	[17]
43*	$HOCl + {}^{\bullet}OH \rightarrow ClO^{\bullet} + H_2O$	2.0×10^9 $M^{-1}s^{-1}$	[13]
44*	$HOCl + O_2^{\bullet-} \rightarrow Cl^{\bullet} + OH^- + O_2$	7.5×10^6 $M^{-1}s^{-1}$	[13]
45*	$HOCl + HO_2^{\bullet} \rightarrow Cl^{\bullet} + OH^- + O_2$	7.5×10^6 $M^{-1}s^{-1}$	[13]
46	$HOCl + NO_2^- \rightleftarrows ClNO_2 + OH^-$	$K_{eq} = 1.1\times10^{-4}$	[18]
47	$ClNO_2 + NO_2^- \rightarrow N_2O_4 + Cl^-$	1.3×10^4 $M^{-1}s^{-1}$	[18]
48*	$OCl^- + {}^{\bullet}OH \rightarrow ClO^{\bullet} + OH^-$	8.8×10^9 $M^{-1}s^{-}$	[13]
49*	$OCl^- + O_2^{\bullet-} + H_2O \rightarrow Cl^{\bullet} + 2OH^- + O_2$	7.5×10^6 $M^{-1}s^{-}$	[13]
50*	$OCl^- + {}^{\bullet}OH \rightarrow ClO_2^{-} + H^+$	$>1\times10^9$ $M^{-1}s^{-1}$	[15]
51	$Cl^- + O_3 \rightarrow ClO^- + O_2$	$<3\times10^3$ $M^{-1}s^{-1}$	[19]
52	$OCl^- + O_3 \rightarrow ClO_2^- + O_2$	30 $M^{-1}s^{-1}$	[15]
53	$OCl^- + O_3 \rightarrow Cl_2 + 2O_2$	110 $M^{-1}s^{-1}$	[15]

54	$ClO_2^- + O_3 \rightarrow ClO_3^- + O_2$	4×10^{6} M^{-1}s^{-1}	[15]
55*	$ClO_2^- + {}^{\bullet}OH \rightarrow ClO_2^{\bullet} + OH^-$	6×10^{9} M^{-1}s^{-1}	[15]
56	$HSO_5^- + Cl^- \rightarrow HOCl + SO_4^{2-}$	2.1×10^{-3} M^{-1}s^{-1}	[20]
Bromide reactions			
57*	$Br^- + {}^{\bullet}OH \rightarrow BrOH^{\bullet -}$	1.1×10^{10} M^{-1}s^{-1}	[13]
58	$Br^- + O_3 \rightarrow BrO^- + O_2$	160 M^{-1}s^{-1}	[21]
59	$Br^- + SO_4^{\bullet -} \rightarrow Br^{\bullet} + SO_4^{2-}$	3.5×10^{9} M^{-1} s^{-1}	[22]
60	$Br^- + NO_3^{\bullet} \rightarrow Br^{\bullet} + NO_3^-$	4×10^{9} M^{-1} s^{-1}	[23]
61*	$Br^{\bullet} + H_2O \rightarrow BrOH^{\bullet -} + H^+$	1.4 M^{-1}s^{-1}	[5]
62*	$Br^{\bullet} + OH^- \rightarrow BrOH^{\bullet -}$	1.6×10^{10}M^{-1}s^{-1}	[24]
63*	$BrOH^{\bullet -} \rightarrow {}^{\bullet}OH + Br^-$	3.3×10^{7} M^{-1}s^{-1}	[24]
64*	$BrOH^{\bullet -} \rightarrow Br^{\bullet} + OH^-$	4.2×10^{6}M^{-1}s^{-1}	[24]
65*	$BrOH^{\bullet -} + H^+ \rightarrow Br^{\bullet} + H_2O$	4.4×10^{10}M^{-1}s^{-1}	[24]
66*	$BrOH^{\bullet -} + Br^- \rightarrow Br_2^{\bullet -} + OH^-$	1.9×10^{8} M^{-1}s^{-1}	[24]
67*	$Br^{\bullet} + Br^- \rightarrow Br_2^{\bullet -}$	1.2×10^{10} M^{-1}s^{-1}	[13]
68*	$Br^{\bullet} + Br^{\bullet} \rightarrow Br_2$	1.0×10^{9} M^{-1}s^{-1}	[13]
69*	$Br^{\bullet} + H_2O_2 \rightarrow HO_2^{\bullet} + Br^-$	4.0×10^{9} M^{-1}s^{-1}	[13]
70*	$Br^{\bullet} + HO_2^{\bullet} \rightarrow H^+ + O_2 + Br^-$	1.0×10^{9} M^{-1}s^{-1}	[13]
71	$Br^{\bullet} + O_3 \rightarrow BrO^{\bullet} + O_2$	1.5×10^{8} M^{-1}s^{-1}	[21]
72*	$Br_2^{\bullet -} \rightarrow Br^{\bullet} + Br^-$	1.9×10^{4} M^{-1}s^{-1}	[13]

73*	$Br_2^{\bullet -}+Br_2^{\bullet -} \rightarrow Br_2+2Br^-$	1.9×10^9 M^{-1}s^{-1}	[13]
74*	$Br_2^{\bullet -}+Br^{\bullet} \rightarrow Br_2+Br^-$	2.0×10^9 M^{-1}s^{-1}	[13]
75*	$Br_2^{\bullet -}+H_2O_2 \rightarrow HO_2^{\bullet}+2Br^- + H^+$	5.0×10^2 M^{-1}s^{-1}	[13]
76*	$Br_2^{\bullet -}+HO_2^{\bullet} \rightarrow O_2+2Br^- + H^+$	1.0×10^8 M^{-1}s^{-1}	[25]
77*	$Br_2^{\bullet -}+HO_2^{\bullet} \rightarrow HO_2^-+2Br^-$	4.4×10^9 M^{-1}s^{-1}	[13]
78*	$Br_2^{\bullet -}+O_2^{\bullet -} \rightarrow O_2+2Br^-$	1.7×10^8 M^{-1}s^{-1}	[25]
79*	$Br_2^{\bullet -}+OBr^- \rightarrow BrO^{\bullet}+2Br^-$	6.2×10^7 M^{-1}s^{-1}	[13]
80*	$Br_2^{\bullet -}+{}^{\bullet}OH \rightarrow HOBr+Br^-$	1.0×10^9 M^{-1}s^{-1}	[25]
81*	$Br_2^{\bullet -}+OH^- \rightarrow BrOH^{\bullet -}+Br^-$	2.7×10^6 M^{-1}s^{-1}	[26]
82*	$Br_2+Br^- \rightarrow Br_3^-$	9.6×10^8 M^{-1}s^{-1}	[13]
83*	$Br_2+HO_2^{\bullet} \rightarrow Br_2^{\bullet -}+O_2 + H^+$	1.1×10^8 M^{-1}s^{-1}	[13]
84*	$Br_2+O_2^{\bullet -} \rightarrow Br_2^{\bullet -} + O_2$	5.6×10^9 M^{-1}s^{-1}	[13]
85*	$Br_2+H_2O \rightarrow HOBr + O_2$	97 M^{-1}s^{-1}	[13]
86*	$Br_2+H_2O_2 \rightarrow 2HBr + O_2$	1.3×10^3 M^{-1}s^{-1}	[25]
87*	$Br_3^- \rightarrow Br_2+Br^-$	5.5×10^7 M^{-1}s^{-1}	[13]
88*	$Br_3^-+HO_2^{\bullet} \rightarrow Br_2^{\bullet -}+HBr + O_2$	1.0×10^7 M^{-1}s^{-1}	[13]
89*	$Br_3^-+O_2^{\bullet -} \rightarrow Br_2^{\bullet -}+Br^- + O_2$	3.8×10^9 M^{-1}s^{-1}	[13]
90*	$BrO^{\bullet} + BrO^{\bullet} \rightarrow BrO_2^- + OBr^-$	5×10^9 M^{-1}s^{-1}	[27]
91*	$2BrO^{\bullet} + H_2O \rightarrow BrO_2^- + OBr^- + 2H^+$	4.9×10^9 M^{-1}s^{-1}	[28]
92*	$BrO^{\bullet} + BrO_2^- \rightarrow OBr^-+BrO_2^{\bullet}$	3.4×10^8 M^{-1}s^{-1}	[28]

93*	$2BrO_2^{\bullet} \rightarrow Br_2O_4$	1.4×10^9 M^{-1}s^{-1}	[28]
94*	$Br_2O_4 \rightarrow 2BrO_2^{\bullet}$	7.4×10^7 M^{-1}s^{-1}	[28]
95*	$BrO_2^{\bullet}+Br_2^{\bullet-} \rightarrow Br^- + BrO^{\bullet} + OBr^-$	8.0×10^7 M^{-1}s^{-1}	[28]
96*	$2BrO_2^{\bullet} + H_2O \rightarrow BrO_2^- + BrO_3^- + 2H^+$	4.2×10^7 M^{-1}s^{-1}	[28]
97*	$HOBr \rightleftarrows BrO^- + H^+$	K$_{eq}$ = 1.58×10^{-9}	[29]
98*	$HOBr + Br^- + H^+ \rightarrow Br_2 + H_2O$	5.0×10^9 M^{-1}s^{-1}	[30]
99*	$HOBr + HO_2^- \rightarrow Br^- + H_2O + O_2$	7.6×10^8 M^{-1}s^{-1}	[31]
100*	$HOBr + H_2O_2 \rightarrow HBr + H_2O + O_2$	1.5×10^4 M^{-1}s^{-1}	[31]
101*	$HOBr + {}^{\bullet}OH \rightarrow BrO^{\bullet} + H_2O$	2.0×10^9 M^{-1}s^{-1}	[13]
102*	$HOBr + O_2^{\bullet-} \rightarrow BrOH^{\bullet-}$	3.5×10^9 M^{-1}s^{-1}	[31]
103*	$HOBr + HO_2^{\bullet} \rightarrow BrOH^{\bullet-} + H^+$	3.5×10^9 M^{-1}s^{-1}	[13]
104*	$HOBr + Br^{\bullet} \rightarrow BrO^{\bullet} + H^+ + Br^-$	5×10^7 M^{-1}s^{-1}	[28]
105	$HOBr + NO_2^- \rightleftarrows BrNO_2 + OH^-$	K$_{eq}$ = 5.6×10^{-6}	[32]
106	$BrNO_2 + NO_2^- \rightarrow N_2O_4 + H^+$	1.4×10^4 M^{-1}s^{-1}	[32]
107*	$OBr^- + Br^{\bullet} \rightarrow BrO^{\bullet} + Br^-$	4.1×10^9 M^{-1}s^{-1}	[28]
108*	$OBr^- + H_2O_2 \rightarrow Br^- + H_2O + O_2$	1.2×10^6 M^{-1}s^{-1}	[31]
109*	$OBr^- + {}^{\bullet}OH \rightarrow BrO^{\bullet} + OH^-$	4.5×10^9 M^{-1}s^{-1}	[13]
110*	$OBr^- + O_2^{\bullet-} + H_2O \rightarrow Br^{\bullet} + 2OH^- + O_2$	2.0×10^8 M^{-1}s^{-1}	[13]
111	$OBr^- + O_3 \rightarrow BrO_2^- + O_2$	100 M^{-1}s^{-1}	[21]
112	$BrO_2^- + O_3 \rightarrow BrO_3^- + O_2$	8.9×10^4 M^{-1}s^{-1}	[21]

113*	$BrO_2^- + Br_2^{\bullet-} \rightarrow Br^- + BrO^\bullet + OBr^-$	8.0×10^7 M^{-1}s^{-1}	[28]
114	$BrO_2^- + O_3 \rightarrow BrO_3^- + O_2$	> 1×10^5 M^{-1} s^{-1}	[21]
115	$Br^- + HSO_5^- \rightarrow HOBr + SO_4^{2-}$	0.7 M^{-1} s^{-1}	[20]
Iodide reactions			
116	$I^- + {}^\bullet OH \rightarrow HOI^{\bullet-}$	1.1×10^{10} M^{-1}s^{-1}	[33]
117	$HOI + HOI \rightarrow IO_2^- + I^- + 2H^+$	0.3 M^{-1}s^{-1}	[34]
118	$HOI + OI^- \rightarrow IO_2^- + I^- + H^+$	15 M^{-1}s^{-1}	[34]
119	$HOI + HOI + HCO_3^- \rightarrow IO_2^- + I^- + 2H^+ + HCO_3^-$	50 M^{-2}s^{-1}	[34]
120	$HOI + HOI + CO_3^{2-} \rightarrow IO_2^- + I^- + 2H^+ + CO_3^{2-}$	5000 M^{-2}s^{-1}	[34]
121	$HOI + HOI + B(OH)_4^- \rightarrow IO_2^- + I^- + 2H^+ + B(OH)_4^-$	1700 M^{-2}s^{-1}	[34]
122	$I^- + HOI + H^+ \rightleftarrows I_2 + H_2O$	K=1.84×10^{12}	[34]
123	$I^- + I_2 \rightleftarrows I_3^-$	K=724	[34]
124	$I^- + O_3 \rightarrow OI^- + O_2$	2×10^9 M^{-1}s^{-1}	[21]
125	$OI^- + 2O_3 \rightarrow IO_3^- + 2O_2$	1.6×10^6 M^{-1}s^{-1}	[21]
126	$HOI + 2O_3 \rightarrow IO_3^- + 2O_2 + H^+$	3.6×10^4 M^{-1}s^{-1}	[21]
127	$I^- + HSO_5^- \rightarrow HOI + SO_4^{2-}$	1.1×10^3 M^{-1} s^{-1}	[20]
128	$I^- + SO_5^{2-} \rightarrow OI^- + SO_4^{2-}$	218 M^{-1} s^{-1}	[35]
129	$HOI + HSO_5^- \rightarrow IO_2^- + SO_4^{2-} + 2H^+$	112 M^{-1} s^{-1}	[35]
130	$OI^- + HSO_5^- \rightarrow IO_2^- + SO_4^{2-} + H^+$	1.7×10^6 M^{-1} s^{-1}	[35]
131	$OI^- + SO_5^{2-} \rightarrow IO_2^- + SO_4^{2-}$	1.5×10^5 M^{-1} s^{-1}	[35]
Mixed halide reactions: Chloride and bromide			
132*	$HOBr + Cl^- \rightarrow BrCl + OH^-$	44 M^{-1}s^{-1}	[36]

133*	$HOCl + Br^{-} \rightarrow BrCl + OH^{-}$	1.0×10^{-2}M^{-1}s^{-1}	[36]
134*	$BrCl + H_2O \rightarrow HOBr + Cl^{-} + H^{+}$	1.0×10^{5}M^{-1}s^{-1}	[13]
135*	$BrCl + H_2O_2 \rightarrow HBr + HCl + H_2O$	1.3×10^{4}M^{-1}s^{-1}	[13]
136*	$BrCl + O_2^{\bullet -} \rightarrow BrCl^{\bullet -} + O_2$	4.0×10^{9}M^{-1}s^{-1}	[13]
137*	$BrCl + HO_2^{\bullet} \rightarrow BrCl^{\bullet -} + O_2 + H^{+}$	5.0×10^{8}M^{-1}s^{-1}	[13]
138*	$BrCl + Cl^{-} \rightarrow BrCl_2^{-}$	1.0×10^{6}M^{-1}s^{-1}	[11]
139*	$BrCl_2^{-} \rightarrow BrCl + Cl^{-}$	1.7×10^{5}s^{-1}	[11]
140*	$BrCl + Br^{-} \rightarrow Br_2Cl^{-}$	3.0×10^{8}M^{-1}s^{-1}	[13]
141*	$Br_2Cl^{-} \rightarrow BrCl + Br^{-}$	1.7×10^{4}s^{-1}	[13]
142*	$Br_2 + Cl^{-} \rightarrow Br_2Cl^{-}$	5.0×10^{4}M^{-1}s^{-1}	[13]
143*	$Br_2Cl^{-} \rightarrow Br_2 + Cl^{-}$	3.8×10^{4}s^{-1}	[13]
144*	$Cl_2 + Br^{-} \rightarrow BrCl_2^{-}$	6.0×10^{9}M^{-1}s^{-1}	[11]
145*	$BrCl_2^{-} \rightarrow Cl_2 + Br^{-}$	9.0×10^{3}s^{-1}	[11]
146*	$Br_2Cl^{-} + Cl^{-} \rightarrow BrCl_2^{-} + Br^{-}$	1.0×10^{5}M^{-1}s^{-1}	[13]
147*	$BrCl_2^{-} + Br^{-} \rightarrow Br_2Cl^{-} + Cl^{-}$	3.0×10^{8} M^{-1}s^{-1}	[11]
148*	$ClOH^{\bullet -} + Br^{-} \rightarrow BrCl^{\bullet -} + OH^{-}$	1.0×10^{9} M^{-1}s^{-1}	[13]
149*	$Cl^{\bullet} + Br^{-} \rightarrow BrCl^{\bullet -}$	1.2×10^{10} M^{-1}s^{-1}	[13]
150*	$Cl_2^{\bullet -} + Br^{-} \rightarrow BrCl^{\bullet -} + Cl^{-}$	4.0×10^{9} M^{-1}s^{-1}	[11]
151*	$BrOH^{\bullet -} + Cl^{-} \rightarrow BrCl^{\bullet -} + OH^{-}$	1.9×10^{8} M^{-1}s^{-1}	[13]
152*	$Br^{\bullet} + Cl^{-} \rightarrow BrCl^{\bullet -}$	1.0×10^{8} M^{-1}s^{-1}	[13]

153*	$Br_2^{\bullet-} + Cl^- \rightarrow BrCl^{\bullet-} + Br^-$	4.3×10^{6} $M^{-1}s^{-1}$	[11]
154*	$Br_2^{\bullet-} + Cl_2^{\bullet-} \rightarrow Br_2 + 2Cl^-$	4.0×10^{9} $M^{-1}s^{-1}$	[13]
155*	$BrCl^{\bullet-} + OH^{\bullet} \rightarrow BrCl + OH^-$	1.0×10^{9} $M^{-1}s^{-1}$	[13]
156*	$BrCl^{\bullet-} + HO_2^{\bullet} \rightarrow Br^- + Cl^- + O_2 + H^+$	1.0×10^{9} $M^{-1}s^{-1}$	[13]
157*	$BrCl^{\bullet-} + O_2^{\bullet-} \rightarrow Br^- + Cl^- + O_2$	6.0×10^{8} $M^{-1}s^{-1}$	[13]
158*	$BrCl^{\bullet-} + H_2O_2 \rightarrow Br^- + HCl + HO_2^{\bullet}$	5.0×10^{3} $M^{-1}s^{-1}$	[13]
159*	$BrCl^{\bullet-} + OH^- \rightarrow ClOH^{\bullet-} + Br^-$	3.0×10^{6} $M^{-1}s^{-1}$	[13]
160*	$BrCl^{\bullet-} + OH^- \rightarrow BrOH^{\bullet-} + Cl^-$	2.0×10^{7} $M^{-1}s^{-1}$	[13]
161*	$BrCl^{\bullet-} + BrCl^{\bullet-} \rightarrow Br^- + Cl^- + BrCl$	4.7×10^{9} $M^{-1}s^{-1}$	[13]
162*	$BrCl^{\bullet-} + Cl_2^{\bullet-} \rightarrow 2Cl^- + BrCl$	2.0×10^{9} $M^{-1}s^{-1}$	[13]
163*	$BrCl^{\bullet-} + Br_2^{\bullet-} \rightarrow Br_2 + Cl^- + Br^-$	4.0×10^{9} $M^{-1}s^{-1}$	[13]
164*	$BrCl^{\bullet-} \rightarrow Cl^{\bullet} + Br^-$	1.9×10^{3} $M^{-1}s^{-1}$	[37]
165*	$BrCl^{\bullet-} \rightarrow Cl^- + Br^{\bullet}$	6.1×10^{4} $M^{-1}s^{-1}$	[37]
166*	$BrCl^{\bullet-} + Br^- \rightarrow Br_2^{\bullet-} + Cl^-$	8.0×10^{9} $M^{-1}s^{-1}$	[11]
167*	$BrCl^{\bullet-} + Cl^- \rightarrow Cl_2^{\bullet-} + Br^-$	1.1×10^{2} $M^{-1}s^{-1}$	[11]
168*	$Cl_2Br^- + H_2O \rightleftarrows HOBr + H^+ + 2Cl^-$	$K_{eq} = 3\times10^{-6}$ M^{-3}	[14]
Mixed halide reactions: Chloride and iodide			
169	$HOCl + I^- \rightarrow HOI + Cl^-$	4.3×10^{8} $M^{-1}s^{-1}$	[21]
170	$OCl^- + I^- \rightarrow OI^- + Cl^-$	< 30 $M^{-1}s^{-1}$	[21]
171	$2HOCl + HOI \rightarrow IO_3^- + 2Cl^- + 3H^+$	8.2 $M^{-1}s^{-1}$	[21]
172	$2OCl^- + HOI \rightarrow IO_3^- + 2Cl^- + H^+$	52 $M^{-1}s^{-1}$	[21]

Mixed halide reactions: Bromide and iodide			
173	$OBr^{-}+I^{-}+H^{+}\rightarrow IBr+OH^{-}$	6.8×10^{5} M^{-1}s^{-1}	[38]
174	$HOBr+I^{-}\rightarrow IBr+OH^{-}$	5.0×10^{9} M^{-1}s^{-1}	[38]
175	$IBr+OH^{-}\rightarrow HOI+Br^{-}$	6×10^{9} M^{-1}s^{-1}	[38]
176	$IBr+H_2O\rightarrow HOI+Br^{-}+H^{+}$	8×10^{5} M^{-1}s^{-1}	[38]
177	$IBr+I^{-}\rightarrow I_2+Br^{-}$	2.1×10^{9} M^{-1}s^{-1}	[38]
178	$HOBr+OI^{-}\rightarrow IO_2^{-}+Br^{-}+H^{+}$	1.9×10^{6} M^{-1}s^{-1}	[39]
179	$OBr^{-}+OI^{-}\rightarrow IO_2^{-}+Br^{-}$	1.8×10^{3} M^{-1}s^{-1}	[39]
180	$HOBr/OBr^{-}+IO_2^{-}\rightarrow IO_3^{-}+Br^{-}$	Very fast	[39]
Bicarbonate reactions			
181*	$^{\bullet}OH+CO_3^{2-}\rightarrow CO_3^{\bullet-}+OH^{-}$	3.9×10^{8} M^{-1} s^{-1}	[33]
182*	$^{\bullet}OH+HCO_3^{-}\rightarrow CO_3^{\bullet-}+H_2O$	8.6×10^{6} M^{-1} s^{-1}	[33]
183*	$^{\bullet}OH+CO_3^{\bullet-}\rightarrow product$	3.0×10^{9} M^{-1} s^{-1}	[40]
184*	$O_2^{\bullet-}+CO_3^{\bullet-}\rightarrow CO_3^{2-}+O_2$	6.0×10^{8} M^{-1} s^{-1}	[40]
185*	$CO_3^{\bullet-}+CO_3^{\bullet-}\rightarrow product$	3.0×10^{7} M^{-1} s^{-1}	[40]
186	$S_2O_8^{2-}+CO_3^{\bullet-}\rightarrow CO_3^{2-}+S_2O_8^{\bullet-}$	3.0×10^{7} M^{-1} s^{-1}	[41]
187	$SO_4^{\bullet-}+HCO_3^{-}\rightarrow CO_3^{\bullet-}+HSO_4^{-}$	2.8×10^{6} M^{-1} s^{-1}	[42]
188	$SO_4^{\bullet-}+CO_3^{2-}\rightarrow CO_3^{\bullet-}+SO_4^{2-}$	6.1×10^{6} M^{-1} s^{-1}	[43]
189*	$OCl^{-}+CO_3^{\bullet-}\rightarrow OCl^{\bullet}+CO_3^{2-}$	5.7×10^{5} M^{-1}s^{-1}	[44]
190*	$Cl^{\bullet}+CO_3^{2-}\rightarrow Cl^{-}+CO_3^{\bullet-}$	5.0×10^{8} M^{-1}s^{-1}	[13]
191*	$Cl^{\bullet}+HCO_3^{-}\rightarrow Cl^{-}+CO_3^{\bullet-}+H^{+}$	2.2×10^{8} M^{-1}s^{-1}	[13]

192*	$Cl_2^{\bullet-} + CO_3^{2-} \rightarrow 2Cl^{-} + CO_3^{\bullet-}$	1.6×10^{8} M^{-1} s^{-1}	[13]
193*	$Cl_2^{\bullet-} + HCO_3^{-} \rightarrow 2Cl^{-} + CO_3^{\bullet-} + H^{+}$	8.0×10^{7} M^{-1} s^{-1}	[13]
194*	$Br_2^{\bullet-} + CO_3^{2-} \rightarrow 2Br^{-} + CO_3^{\bullet-}$	1.1×10^{5} M^{-1} s^{-1}	[13]
195*	$Br_2^{\bullet-} + HCO_3^{-} \rightarrow 2Br^{-} + CO_3^{\bullet-} + H^{+}$	8.0×10^{4} M^{-1} s^{-1}	[13]
196*	$Br^{\bullet} + CO_3^{2-} \rightarrow Br^{-} + CO_3^{\bullet-}$	3.4×10^{4} M^{-1} s^{-1}	[44]
197*	$Br^{\bullet} + CO_3^{2-} \rightarrow Br^{-} + CO_3^{\bullet-}$	2.0×10^{6} M^{-1} s^{-1}	[13]
198*	$Br^{\bullet} + HCO_3^{-} \rightarrow Br^{-} + CO_3^{\bullet-} + H^{+}$	1.0×10^{6} M^{-1} s^{-1}	[13]
199*	$BrCl^{\bullet-} + HCO_3^{-} \rightarrow Br^{-} + HCl + CO_3^{\bullet-}$	3.0×10^{6} M^{-1} s^{-1}	[13]
200*	$BrCl^{\bullet-} + CO_3^{2-} \rightarrow Br^{-} + Cl^{-} + CO_3^{\bullet-}$	6.0×10^{6} M^{-1} s^{-1}	[13]
Other reactions in modeling			
201*	$H_2O \rightleftarrows H^{+} + OH^{-}$	$K_{eq} = 1.0\times10^{-14}$	
202*	$H_2O_2 \rightleftarrows H^{+} + HO_2^{-}$	$K_{eq} = 2.5\times10^{-12}$	[29]
203*	$HO_2^{\bullet} \rightarrow H^{+} + O_2^{\bullet-}$	$K_{eq} = 1.6\times10^{-5}$	[45]
204*	$HCO_3^{-} + H^{+} \rightarrow H_2CO_3$	1.0×10^{10} $M^{-1}s^{-1}$	
205*	$H_2CO_3 \rightarrow HCO_3^{-} + H^{+}$	$K_{eq} = 4.5\times10^{-7}$ s^{-1}	[46]
206*	$HCO_3^{-} \rightarrow CO_3^{2-} + H^{+}$	$K_{eq} = 4.4\times10^{-11}$ s^{-1}	[46]
207*	$^{\bullet}OH + ^{\bullet}OH \rightarrow H_2O_2$	$5.5\times10^{10}M^{-1}s^{-1}$	[33]
208*	$^{\bullet}OH + OH^{-} \rightarrow O^{\bullet-} + H_2O$	$1.2\times10^{10}M^{-1}s^{-1}$	[33]
209*	$^{\bullet}OH + H_2O_2 \rightarrow HO_2^{\bullet} + HO_2$	$2.7\times10^{9}M^{-1}s^{-1}$	[33]
210*	$^{\bullet}OH + HO_2^{-} \rightarrow HO_2^{\bullet} + OH^{-}$	7.5×10^{9} $M^{-1}s^{-1}$	[33]

211*	$^{\bullet}OH+HO_2^{\bullet} \rightarrow O_2+H_2O$	$6.6\times10^{9}M^{-1}s^{-1}$	[40]
212*	$^{\bullet}OH+O_2^{\bullet-} \rightarrow O_2+OH^{-}$	7.0×10^{9} $M^{-1}s^{-1}$	[40]
213*	$HO_2^{\bullet}+HO_2^{\bullet} \rightarrow H_2O_2+O_2$	$8.3\times10^{9}M^{-1}s^{-1}$	[33]
214*	$HO_2^{\bullet}+O_2^{\bullet-} \rightarrow HO_2^{-}+O_2$	$7.5\times10^{9}M^{-1}s^{-1}$	[33]
215*	$HO_2^{\bullet}+H_2O_2 \rightarrow O_2+{}^{\bullet}OH+H_2O$	3 $M^{-1}s^{-1}$	[33]
216*	$O_2^{\bullet-}+H_2O_2 \rightarrow O_2+{}^{\bullet}OH+OH^{-}$	7.5×10^{9} $M^{-1}s^{-1}$	[33]
217*	$H_2O_2+CO_3^{\bullet-} \rightarrow HCO_3^{-}+HO_2^{\bullet}$	4.5×10^{5} $M^{-1}s^{-1}$	[47]
218*	$HO_2^{-}+CO_3^{\bullet-} \rightarrow CO_3^{2-}+HO_2^{\bullet}$	3.0×10^{7} $M^{-1}s^{-1}$	[47]

* Reactions in modeling (163 in total).

REFERENCES

1. Jayson, G. G.; Parsons, B. J.; Swallow, A. J., Some Simple, Highly Reactive, Inorganic Chlorine Derivatives in Aqueous-Solution - Their Formation Using Pulses of Radiation and Their Role in Mechanism of Fricke Dosimeter. *J Chem Soc Farad T 1* **1973**, (9), 1597-1607.
2. Das, T. N., Reactivity and role of SO5•- radical in aqueous medium chain oxidation of sulfite to sulfate and atmospheric sulfuric acid generation. *J. Phys. Chem. A* **2001,** 105, (40), 9142-9155.
3. Poskrebyshev, G. A.; Huie, R. E.; Neta, P., The Rate and Equilibrium Constants for the Reaction NO3• + Cl- ⇄ NO3- + Cl• in Aqueous Solutions. *J. Phys. Chem. A* **2003,** 107, (12), 1964-1970.
4. Grigorev, A. E.; Makarov, I. E.; Pikaev, A. K., Formation of Cl2- in the Bulk Solution during the Radiolysis of Concentrated Aqueous-Solutions of Chlorides. *High Energ. Chem.* **1987,** 21, (2), 99-102.
5. Klaning, U. K.; Wolff, T., Laser Flash-Photolysis of Hclo, Clo-, Hbro, and Bro- in Aqueous-Solution - Reactions of Cl-Atoms and Br-Atoms. *PCCP* **1985,** 89, (3), 243-245.
6. Yu, X. Y.; Barker, J. R., Hydrogen peroxide photolysis in acidic aqueous solutions containing chloride ions. II. Quantum yield of HO center dot(Aq) radicals. *J. Phys. Chem. A* **2003,** 107, (9), 1325-1332.
7. Wu, D.; Wong, D.; Dibartolo, B., Evolution of Cl2- in Aqueous Nacl Solutions. *J. Photochem.* **1980,** 14, (4), 303-310.
8. Technology, N. I. o. S. a., NDRL/NIST Solution Kinetics Database on the Web. http://kinetics.nist.gov/solution/ **2002**.
9. Neta, P.; Huie, R. E.; Ross, A. B., Rate constants for reactions of inorganic radicals in aqueous solution. *J. Phys. Chem. Ref. Data* **1988,** 17, (3), 1027-1284.
10. Mcelroy, W. J., A Laser Photolysis Study of the Reaction of So4- with Cl- and the Subsequent Decay of Cl2- in Aqueous-Solution. *J. Phys. Chem.* **1990,** 94, (6), 2435-2441.
11. Ershov, B. G., Kinetics, mechanism and intermediates of some radiation-induced reactions in aqueous solutions. *Usp. Khim.* **2004,** 73, (1), 107-120.

12. Bjergbakke, E.; Navaratnam, S.; Parsons, B. J.; Swallow, A. J., Reaction between Ho2 and Chlorine in Aqueous-Solution. *J. Am. Chem. Soc.* **1981,** 103, (19), 5926-5928.
13. Matthew, B. M.; Anastasio, C., A chemical probe technique for the determination of reactive halogen species in aqueous solution: Part 1 - bromide solutions. *Atmos. Chem. Phys.* **2006,** 6, 2423-2437.
14. Wang, T. X.; Margerum, D. W., Kinetics of Reversible Chlorine Hydrolysis - Temperature-Dependence and General Acid Base-Assisted Mechanisms. *Inorg. Chem.* **1994,** 33, (6), 1050-1055.
15. Siddiqui, M. S., Chlorine-ozone interactions: Formation of chlorate. *Water Res.* **1996,** 30, (9), 2160-2170.
16. Maetzke, A.; Knak Jensen, S. J., Reaction paths for production of singlet oxygen from hydrogen peroxide and hypochlorite. *Chem. Phys. Lett.* **2006,** 425, (1–3), 40-43.
17. Connick, R. E., The Interaction of Hydrogen Peroxide and Hypochlorous Acid in Acidic Solutions Containing Chloride Ion. *J. Am. Chem. Soc.* **1947,** 69, (6), 1509-1514.
18. Lahoutifard, N.; Lagrange, P.; Lagrange, J., Kinetics and mechanism of nitrite oxidation by hypochlorous acid in the aqueous phase. *Chemosphere* **2003,** 50, (10), 1349-1357.
19. Hoigné, J.; Bader, H.; Haag, W. R.; Staehelin, J., Rate constants of reactions of ozone with organic and inorganic compounds in water—III. Inorganic compounds and radicals. *Water Res.* **1985,** 19, (8), 993-1004.
20. Lente, G.; Kalmár, J.; Baranyai, Z.; Kun, A.; Kék, I.; Bajusz, D.; Takács, M.; Veres, L.; Fábián, I., One- Versus Two-Electron Oxidation with Peroxomonosulfate Ion: Reactions with Iron(II), Vanadium(IV), Halide Ions, and Photoreaction with Cerium(III). *Inorg. Chem.* **2009,** 48, (4), 1763-1773.
21. von Gunten, U., Ozonation of drinking water: Part II. Disinfection and by-product formation in presence of bromide, iodide or chlorine. *Water Res.* **2003,** 37, (7), 1469-1487.
22. Peyton, G. R., The free-radical chemistry of persulfate-based total organic-carbon analyzers. *Mar. Chem.* **1993,** 41, (1-3), 91-103.
23. Neta, P.; Huie, R. E., Rate constants for reactions of nitrogen oxide (NO3) radicals in aqueous solutions. *J. Phys. Chem.* **1986,** 90, (19), 4644-4648.
24. Zehavi, D.; Rabani, J., Oxidation of Aqueous Bromide Ions by Hydroxyl Radicals - Pulse Radiolytic Investigation. *J. Phys. Chem.* **1972,** 76, (3), 312-319.
25. Wagner, I.; Strehlow, H., On the Flash-Photolysis of Bromide Ions in Aqueous-Solutions. *PCCP* **1987,** 91, (12), 1317-1321.
26. Mamou, A.; Rabani, J.; Behar, D., Oxidation of Aqueous Br- by Oh Radicals, Studied by Pulse-Radiolysis. *J. Phys. Chem.* **1977,** 81, (15), 1447-1448.
27. Pinkernell, U.; von Gunten, U., Bromate minimization during ozonation: Mechanistic considerations. *Environ. Sci. Technol.* **2001,** 35, (12), 2525-2531.
28. Lutze, H. V.; Bakkour, R.; Kerlin, N.; von Sonntag, C.; Schmidt, T. C., Formation of bromate in sulfate radical based oxidation: Mechanistic aspects and suppression by dissolved organic matter. *Water Res.* **2014,** 53, (0), 370-377.
29. Von Gunten, U.; Oliveras, Y., Kinetics of the reaction between hydrogen peroxide and hypobromous acid: Implication on water treatment and natural systems. *Water Res.* **1997,** 31, (4), 900-906.
30. Eigen, M.; Kustin, K., Kinetics of Halogen Hydrolysis. *J. Am. Chem. Soc.* **1962,** 84, (8), 1355-1361.
31. VonGunten, U.; Oliveras, Y., Kinetics of the reaction between hydrogen peroxide and hypobromous acid: Implication on water treatment and natural systems. *Water Res.* **1997,** 31, (4), 900-906.

32. Lahoutifard, N.; Lagrange, P.; Lagrange, J.; Scott, S. L., Kinetics and Mechanism of Nitrite Oxidation by HOBr/BrO- in Atmospheric Water and Comparison with Oxidation by HOCl/ClO. *J. Phys. Chem. A* **2002,** 106, (49), 11891-11896.
33. Buxton, G. V.; Greenstock, C. L.; Helman, W. P.; Ross, A. B., Critical review of rate constants for reactions of hydrated electrons, hydrogen atoms and hydroxyl radicals. *J. Phys. Chem. Ref. Data* **1988,** 17, (2), 513-886.
34. Bichsel, Y.; von Gunten, U., Hypoiodous acid: kinetics of the buffer-catalyzed disproportionation. *Water Res.* **2000,** 34, (12), 3197-3203.
35. Li, J.; Jiang, J.; Zhou, Y.; Pang, S.-Y.; Gao, Y.; Jiang, C.; Ma, J.; Jin, Y.; Yang, Y.; Liu, G.; Wang, L.; Guan, C., Kinetics of Oxidation of Iodide (I–) and Hypoiodous Acid (HOI) by Peroxymonosulfate (PMS) and Formation of Iodinated Products in the PMS/I–/NOM System. *Environmental Science & Technology Letters* **2017,** 4, (2), 76-82.
36. Sander, R.; Vogt, R.; Harris, G. W.; Crutzen, P. J., Modelling the chemistry of ozone, halogen compounds, and hydrocarbons in the arctic troposphere during spring. *Tellus B* **1997,** 49, (5), 522-532.
37. Donati, A. Spectroscopic and Kinetic Investigations of Halogen Containing Radicals in the Tropospheric Aqueous Phase. University of Leipzig, Leipzig, Germany, 2002.
38. Troy, R. C.; Margerum, D. W., Non-metal redox kinetics: Hypobromite and hypobromous acid reactions with iodide and with sulfite and the hydrolysis of bromosulfate. *Inorg. Chem.* **1991,** 30, (18), 3538-3543.
39. Criquet, J.; Allard, S.; Salhi, E.; Joll, C. A.; Heitz, A.; von Gunten, U., Iodate and Iodo-Trihalomethane Formation during Chlorination of Iodide-Containing Waters: Role of Bromide. *Environ. Sci. Technol.* **2012,** 46, (13), 7350-7357.
40. Crittenden, J. C.; Hu, S.; Hand, D. W.; Green, S. A., A kinetic model for H2O2/UV process in a completely mixed batch reactor. *Water Res.* **1999,** 33, (10), 2315-2328.
41. Yang, Y.; Pignatello, J. J.; Ma, J.; Mitch, W. A., Comparison of halide impacts on the efficiency of contaminant degradation by sulfate and hydroxyl radical-based advanced oxidation processes (AOPs). *Environ. Sci. Technol.* **2014,** 48, (4), 2344-2351.
42. Huie, R. E.; Clifton, C. L., Temperature dependence of the rate constants for reactions of the sulfate radical, SO4-, with anions. *J. Phys. Chem.* **1990,** 94, (23), 8561-8567.
43. Zuo, Z. H.; Cai, Z. L.; Katsumura, Y.; Chitose, N.; Muroya, Y., Reinvestigation of the acid-base equilibrium of the (bi)carbonate radical and pH dependence of its reactivity with inorganic reactants. *Radiat. Phys. Chem.* **1999,** 55, (1), 15-23.
44. Huie, R. E.; Clifton, C. L.; Neta, P., Electron-transfer reaction-rates and equilibria of the carbonate and sulfate radical-anions. *Radiat. Phys. Chem.* **1991,** 38, (5), 477-481.
45. National Insititute of Standards and Technology NDRL/NIST Solution Kinetics Database on the Web. http://kinetics.nist.gov/solution/ (accessed August 27, 2013),
46. Acero, J. L.; von Gunten, U., Influence of carbonate on the ozone/hydrogen peroxide based advanced oxidation process for drinking water treatment. *Ozone-Sci Eng* **2000,** 22, (3), 305-328.
47. Draganic, Z. D.; Negronmendoza, A.; Sehested, K.; Vujosevic, S. I.; Navarrogonzales, R.; Albarransanchez, M. G.; Draganic, I. G., Radiolysis of Aqueous-Solutions of Ammonium Bicarbonate over a Large Dose Range. *Radiat. Phys. Chem.* **1991,** 38, (3), 317-321.
